# Landscape Genetics for the Empirical Assessment of Resistance Surfaces: The European Pine Marten (*Martes martes*) as a Target-Species of a Regional Ecological Network

**DOI:** 10.1371/journal.pone.0110552

**Published:** 2014-10-16

**Authors:** Aritz Ruiz-González, Mikel Gurrutxaga, Samuel A. Cushman, María José Madeira, Ettore Randi, Benjamin J. Gómez-Moliner

**Affiliations:** 1 Department of Zoology and Animal Cell Biology, University of the Basque Country, UPV/EHU, Vitoria-Gasteiz, Spain; 2 Systematics, Biogeography and Population Dynamics Research Group, Lascaray Research Center, University of the Basque Country, UPV/EHU, Vitoria-Gasteiz, Spain; 3 Conservation Genetics Laboratory, National Institute for Environmental Protection and Research, ISPRA, Ozzano dell'Emilia, Bologna, Italy; 4 Department of Geography, University of the Basque Country, UPV/EHU, Vitoria-Gasteiz, Spain; 5 U.S. Forest Service, Rocky Mountain Research Station, Flagstaff, AZ, United States of America; 6 Department 18/Section of Environmental Engineering, Aalborg University, Aalborg, Denmark; Smithsonian Conservation Biology Institute, United States of America

## Abstract

Coherent ecological networks (EN) composed of core areas linked by ecological corridors are being developed worldwide with the goal of promoting landscape connectivity and biodiversity conservation. However, empirical assessment of the performance of EN designs is critical to evaluate the utility of these networks to mitigate effects of habitat loss and fragmentation. Landscape genetics provides a particularly valuable framework to address the question of functional connectivity by providing a direct means to investigate the effects of landscape structure on gene flow. The goals of this study are (1) to evaluate the landscape features that drive gene flow of an EN target species (European pine marten), and (2) evaluate the optimality of a regional EN design in providing connectivity for this species within the Basque Country (North Spain). Using partial Mantel tests in a reciprocal causal modeling framework we competed 59 alternative models, including isolation by distance and the regional EN. Our analysis indicated that the regional EN was among the most supported resistance models for the pine marten, but was not the best supported model. Gene flow of pine marten in northern Spain is facilitated by natural vegetation, and is resisted by anthropogenic landcover types and roads. Our results suggest that the regional EN design being implemented in the Basque Country will effectively facilitate gene flow of forest dwelling species at regional scale.

## Introduction

Long-term biodiversity conservation requires the preservation of ecological and evolutionary processes, such as gene flow, dispersal movements and population range shifts [1]. The ability of individuals to move across changing landscapes is crucial for maintaining regional populations [2], [3]. The preservation of these processes requires, in turn, that landscape connectivity be preserved, especially when we take into account the synergetic effects of habitat fragmentation and climate change [1]. Landscape connectivity is defined as the degree to which landscape facilitates or impedes movement of organisms among resource patches [4]. Connectivity is species-specific and reflects the response of individuals to landscape features and the patterns of dispersal and gene flow that result from these individual responses [5]. Thus, landscape connectivity depends to a large extent on how the spatial configuration of habitat and land use interact with the movement ecology of particular species [6].

Ecological networks have been promoted as coherent systems composed of core areas linked by ecological corridors capable of facilitating the dispersal, migration and gene flow of wild species in landscapes and regions [7]–[9]. They are configured and managed with the objective of maintaining ecological functions and conserving biodiversity [7]. Although the development of ecological networks is based on the precautionary principle and on ecological theory [8], the absence of empirical evidence regarding their effectiveness and the difficulty in obtaining this evidence has been a focus of criticism about the extent to which they have in fact ensured landscape connectivity and increased biodiversity conservation [10], [11].

In the design of ecological networks there is a need to predict regional ecological corridors and to quantify the degree of expected landscape connectivity between specific areas [3], [9], [11]–[13]. ‘Least-cost modeling’ is one commonly employed approach for designing ecological corridors [9], [14], in which resistance values are assigned to distinct habitat or land use types and the least-cost paths (LCP) between specific locations are calculated using a geographical information system (GIS). How landscape influences effective distances between locations is calculated as the accumulated cost through the least cost paths [14], [15]. However, for most organisms, setting the resistance values is a difficult process in which expert judgment and data available in the literature play an important role [16]–[19].

Accurate identification of the potential factors that drive gene flow in heterogenous landscapes and the scales at which they are acting is a foundation of reliable mapping of corridors [9], [18]. Thus, reliable development of corridors must be based on a correct representation of the local resistance relative to the movement ecology of the organism of focus [9], [18]. Landscape genetics, a research area that integrates landscape ecology, population genetics and spatial statistics, provides a valuable framework for testing the influence of landscape structure and composition on dispersal and gene flow [20], [21]. It facilitates quantification of the resistance to gene flow a given landscape element poses [12], [22]. Thus, one of the principal applications of landscape genetics in landscape planning and conservation biology is to empirically test and optimize resistance maps [23]–[26]. This facilitates the optimal design of ecological corridors [3], [16], [23], the detection of barriers to gene flow [27]–[29] and the identification of the landscape features which favour or impede dispersal [30]–[35].

Landscape genetics has shifted towards individual-based sampling and analysis, especially when organisms are continuously distributed [12], [22]. However, sufficient sample collection for this purpose is a difficult task, especially in rare and elusive species in which sampling is a limiting factor [36]. In this context, non-invasive genetic sampling allows us to address studies of wildlife species without the need to capture or even observe them [37]–[40].

In 2005 a regional ecological network was established in the Basque Country (North Spain) by delimiting the ecological corridors linking forest protected areas [41]. A functional group of forest mammal species was selected to guide the development of a generic resistance map, which would, in turn, serve as a basis least-cost modeling of the network of ecological corridors linking these core areas. These mammals were considered suitable target species due to their sensitivity to recent fragmentation and homogenization dynamics in the regional landscape, such as road construction, urbanization and agrarian intensification [41], [42]. The resistance map was parameterized through bibliographical review and expert opinion and was based on the assignment of different resistance levels to each land use [41]. The regional government of the Basque country incorporated that coherent ecological network as a reference for the environmental assessment of plans, programs and projects in 2005 [41]. In addition to its intrinsic internal relevance, the Basque country has been chosen for its crucial role in the regulation of biotic flows in south-western Europe [43]. This is because of its strategic location between two important biodiversity reservoirs in south-western Europe, the mountain chains of the Pyrenees and the Cantabrian Range [43]–[45]. Consequently, the preservation and restoration of connectivity in this transitional area between mountain ranges requires reliable knowledge about ecological responses of organisms to landscape composition and structure [45].

Among the set of functional forest mammals used in the design of the coherent regional ecological network, the European pine marten (*Martes martes*) is the most forest dependent species [46]. The pine marten is generally associated with forest habitats, mainly mature forests [46]–[48]. Deforestation and forest fragmentation limit the distribution and density of pine martens [48]–[50], which are believed to need a minimum woodland area to survive (ca. 2km^2^) [47] and tend to avoid treeless areas [48], [51], [52]. Their occurrence patterns are affected by forest patch size, percentage of woodland cover, food abundance, sex, age class and habitat fragmentation levels [47], [51]. Given their strong associations with high forest structural complexity, the species is particularly sensitive to human influences on their habitats, including habitat loss and landscape-scale effects of habitat fragmentation [48], [51], [53]. Nonetheless, they have also been recently reported in fragmented landscapes characterized by isolated, small forest fragments within an agricultural landscape matrix [48], [51], [54], suggesting they are not as obligately interior-forest dependent as previously described [51]. However, in such landscapes, linear features, such as hedgerows and small woods, play a key role to connect adjacent forest patches [48], [54], [55].

Consequently, the pine marten is a species which is well suited to studies focused on the effects of forest fragmentation on genetic structure and gene flow [56]. However, whether habitat characteristics that predict marten occupancy act as barriers to dispersal, influencing gene flow and population genetic structure across the landscape, is largely unknown [56].

The main objective of this research is to evaluate a large suite of alternative resistance hypotheses for the pine marten and compare the most supported empirical model with the expert-derived landscape resistance model used to parameterize the corridor network for the Basque Country. Specifically, we aim to evaluate (1) different binary landscape resistance maps which cover a gradient from greater to lesser preference of the pine marten for forest environments in order to identify which land uses favour or impede genetic interchange in the study area; and secondly (2) whether or not the resistance map with which the regional ecological network was originally designed in the Basque Country was correctly parametrized to reflect European pine marten gene flow.

If there is no effect of landscape structure on dispersal and gene flow in martens, then we expected either: (a) panmixia, where there is no genetic pattern, or (b) isolation-by-distance, where genetic differences increase with geographic distance [57]. If landscape structure influences marten dispersal, then we expected (c) isolation by landscape resistance [30]. Given that the most consistent marten-habitat relation appears to be a general association with forest habitats, and avoidance of open, non-forested habitats [47], [48], [51], [52], we expected that open and human altered landscapes would act as a barrier for martens, and hence that landscape structure would have an effect on gene flow. In addition, we hypothesize that the intervening landscape features between forest patches (i.e., matrix) could also play a key role to substantially affect pine marten dispersal, and consequently the connectivity between forest environments [48], [55], [58].

## Methods

### Study area and spatial data

**Figure 1:**
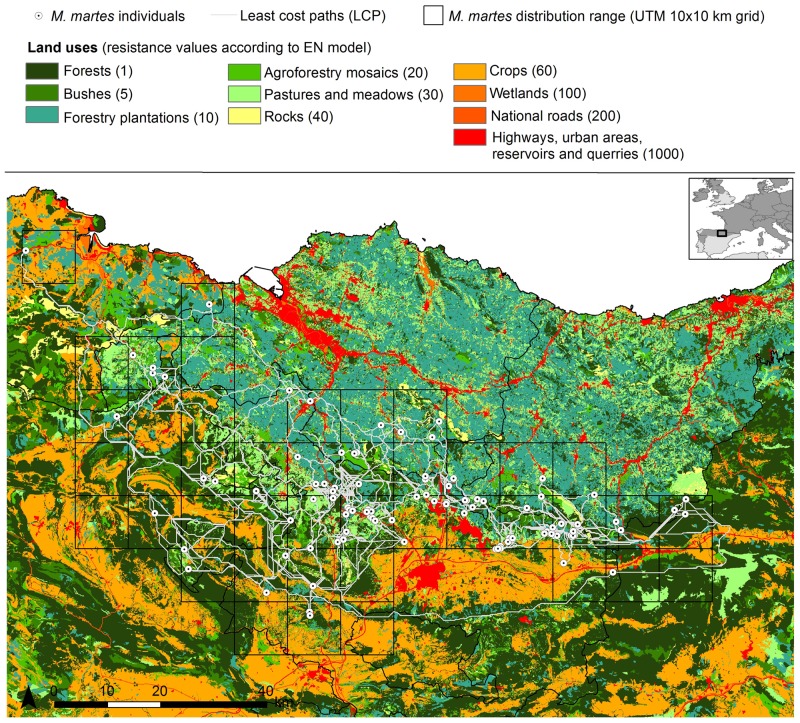
Ecological network resistance map (EN) and LCP analysis between European pine marten individuals in the study area. Least cost paths (LCP) obtained between the 101 pine marten individuals in accordance with the EN resistance map, analogous to that used in the design of the corridors in the ecological network of the Basque Country (North Spain) [41]. Resistance values for each land use are indicated in brackets.

The region of the Basque Country is located in the northern Iberian Peninsula (Fig. 1) within the Atlantic and Mediterranean biogeographical regions. It comprises an area of 7,235 km^2^ and has an average human population density of 298 inhabitants per square kilometer. Forests cover 28%, forestry plantations 29%, non-wooded mountains 24%, cultivated land 14%, and urban land and infrastructures 5.7% of the land area, respectively.

Land use information was obtained in vector format from the most recent forest map of Spain [59] and from national road network maps [60].

### Non-invasive genetic sampling and species identification

We used non-invasive scat sampling to collect genetic samples from the *Martes* sp. (*Martes martes* and *Martes foina*) in the study area between 2004 and 2010. Thus, no specific permissions were required for faecal sampling purposes, as the sampling was carried out without needing to intervene directly in the species in focus. We conducted a multi-stage sampling scheme, in which samples from a pilot study were used to assess the appropriateness of the sampling with respect to the research questions. Thus, two scat-based surveys were conducted between 2004 and 2010 across the sympatric range of both species in the study area. The first survey, conducted in 2004–2005, was used to initially estimate the distribution range of the two sympatric species of the genus *Martes* in the study area and isolate genetic samples of the focal species (*M. martes*). The second, conducted in 2006–2010, was used to refine species distribution information and to obtain a higher number of *M. martes* samples for microsatellite genotyping after a genetic species identification process [52] Aiming to homogenously cover the wide study area and obtain the highest number of different individuals we prioritized our sampling to faecal samples that were separated a minimum of 1km apart (i.e. potentially avoiding re-samplings of the same individual). We also prioritized fresh scat samples to increase genotyping success [61]. Additionally, fresh tissue specimens from road-killed pine martens were included in the data base, when possible. Tissue specimens were collected by authorized veterinarian personnel of the Wildlife Rehabilitation Centre of Martioda (Alava Regional Council. Department of Environment. Biodiversity section), in line with the laws and ethical protocols governing wildlife management (Law 42/2007) and were submitted to Department of Zoology and Animal Cell Biology (UPV/EHU) for further DNA analyses. No animals were sacrificed for the only purposes of this study. Therefore, a formal approval by an Institutional Animal Care and Use Committee was not necessary. Universal Transversal Mercator (UTM) coordinates were recorded for all the samples collected using a global positioning system (Garmin eTtrex) [61]. The faecal samples were stored in autoclaved tubes containing ethanol 96% and frozen at −20°C until processed [52]. DNA was isolated from tissues and scat using the Qiagen DNeasy Tissue DNA (Qiagen, Hombrechtikon, Switzerland) and DNA Stool MiniKit (Qiagen, Hombrechtikon, Switzerland) according to the manufacturer's instructions, respectively. As pine marten faeces cannot be distinguished from those of the sympatric stone marten (*M. foina*), which is widespread in the study area, and can also be easily confused with those of other carnivores [62], a molecular technique was applied for the identification of faecal samples. Species identification was accomplished by a polymerase chain reaction – restriction fragment length polymorphism (PCR-RFLP) method, providing for an effective genetic identification of sympatric marten species following the method described in Ruiz-González et al. [52].

### Microsatellite analyses and individual identification

Identification of individual pine martens used nuclear DNA following methods in Ruiz-González et al. [61]. All the faecal samples identified by the PCR-RFLP method [52] as pine marten were genotyped at 15 variable microsatellite loci (Table S1) using a multiplex protocol specifically designed for degraded faecal DNA analysis [61] and following a modified multitube-approach [63]. The multitube-approach of 4 independent replicates followed by a stringent criteria to construct consensus genotype (i.e. accepting heterozygotes if the two alleles were seen at least in two replicates and homozygotes if a single allele was seen at least in three replicates) is a commonly used approach in non invasive genetic studies leading to a low probability of retaining a false homozygote or false allele error (e.g. [64]–[66]). Briefly, DNA quality was initially screened by PCR-amplifying each DNA sample four times at four loci (Multiplex 1: MP0188; MP0059; Gg-7; Ma-1), since the results obtained for this four loci are indicative of the genotyping success for the full panel of 15 microsatellites [61].

Only samples showing> 50% positive PCRs were further amplified four times at the remaining 11 loci. Samples with ambiguous results after four amplifications per locus or with <50% successful amplifications across loci were removed from further analysis as they were not considered reliable genotypes. Multiplex PCR products were run on an ABI (Foster City, CA) 3130XL automated sequencer (Applied Biosystems), with the internal size standard GS500 LIZ (Applied Biosystems). Fragment analyses were conducted using the ABI software Genemapper 4.0.

RELIOTYPE software [67] was used to assess genotype reliability obtained by 4 independent replicates. Samples that were not reliably typed at all loci after 4 replicates (at score threshold *R* = 0.95) were discarded from the analysis. GIMLET software v 1.3.4 [68] was used to calculate the probabilities of identity (PID and PID-sibs) so as to quantify the efficacy in discriminating the fifteen loci in combination. Consensus genotypes from four replicates were reconstructed using GIMLET, accepting heterozygotes if the two alleles were seen at least in two replicates and homozygotes if a single allele was seen at least in three replicates (e.g. [64]–[66]). GIMLET was also used to estimate genotyping errors: allelic dropout (ADO) and false alleles (FA) [63], [69].

The raw microsatellite data and geographic coordinates of the 101 pine marten individuals are included in Table S2.

### Genetic diversity and pairwise individual genetic distances

We summarized genetic variation through the number of alleles per locus (A), expected (HE) and observed (HO) heterozygosities using GENETIX v 4.05.2 [70]. Estimates of pairwise linkage disequilibria for each pair of loci and deviation from Hardy Weinberg equilibrium (HWE) genotypic proportions at each locus were tested using the exact test implemented in GenePop version 4.0 [71]. Statistical significance was evaluated by running a Markov Chain Monte Carlo (MCMC) consisting of 10,000 batches of 10,000 iterations each, with the first 10,000 iterations discarded before sampling [72]. Significance levels were adjusted with sequential Bonferroni correction in order to correct for the effect of multiple tests [73], (i.e. α = 0.05/number markers). MICRO-CHECKER software [74] was used to check for potential scoring errors and the presence of null alleles. The Rousset's a_r_ inter-individual genetic distance [75] was computed using the program SPAGeDI [76] since this parameter of relatedness does not rely on a reference population [77] and has been successfully applied to infer the effect of landscape on genetic structure of continuously distributed vertebrates [32], [78]–[80].

### Construction of landscape resistance models

We produced different resistance maps representing 59 different hypotheses about the resistance of different land use types using ARCGIS version 9.3 [81], with a raster cell size set to 50 m (Table 1; File S1). As suggested by Anderson et al. [82] the sampling grain selected (i.e. 50×50 m) is adequate to infer landscape effects on gene flow as is smaller than the average home-range size of the study species (i.e.> 0.5Km^2^, [47]). In addition, this resolution allows representation of small landscape patches, but also those smaller elements in the landscape that will be crucial for the resulting effective distances, including linear elements such roads and highways [14].

**Table 1:** Resistance values corresponding to the resistance maps taken evaluated.

		Binary landscape resistance maps														Ecological Network	
		Land_A_x_ to Land_G_x_							Land_A_x_ to Land_G_x_								
Land uses	IBD	A_x_	B_x_	C_x_	D_x_	E_x_	F_x_	G_x_	Ab_x_	Bb_x_	Cb_x_	Db_x_	Eb_x_	Fb_x_	Gb_x_	EN	ENnb
Forests	1	1	1	1	1	1	1	1	1	1	1	1	1	1	1	1	1
Forestry plantations	1	X	1	1	1	1	1	1	X	1	1	1	1	1	1	10	10
Scrubland	1	X	X	1	1	1	1	1	X	X	1	1	1	1	1	5	5
Agroforestry mosaics	1	X	X	X	1	1	1	1	X	X	X	1	1	1	1	20	20
Pastures and meadows	1	X	X	X	X	1	1	1	X	X	X	X	1	1	1	30	30
Rocks	1	X	X	X	X	X	1	1	X	X	X	X	X	X	1	40	40
Crops	1	X	X	X	X	X	X	1	X	X	X	X	X	X	1	60	60
Wetlands	1	X	X	X	X	X	X	X	100	100	100	100	100	100	100	100	100
National roads	1	X	X	X	X	X	X	X	200	200	200	200	200	200	200	200	50
Highways, urban areas, reservoirs and quarries	1	X	X	X	X	X	X	X	1000	1000	1000	1000	1000	1000	1000	1000	50

*Binary landscape resitance maps*: 1) *Land_A-Land_G*: Binary resistance maps, on a gradient from greater to lesser preference of the focal species in relation to forest environment; 2) *Land_Ab-Land_Gb*: Maps with letter “b” include the barrier effect of national roads, highways, urban areas, reservoirs and quarries. All the models were evaluated for 4 different resistance values (X =  5, 25, 50, 100) (e.g. Land_A_5_ correspond to Land_A model with resistance value of 5). *Ecological Network resistance map*: 1) *EN*: a resistance map analogous to that used in the design of the ecological network of the Basque country; *ENnb*: a variant of the latter which diminishes the barrier effect of national roads, highways, urban areas, water reservoirs and quarries.

**1) Isolation by distance:** Our first hypothesis and null model was a test of isolation by distance across a uniform resistance landscape [57], [83]. In this model we assumed movement could occur with equal facility in any direction, with all raster cell values equal in resistance (i.e. resistance value 1).

**Figure 2:**
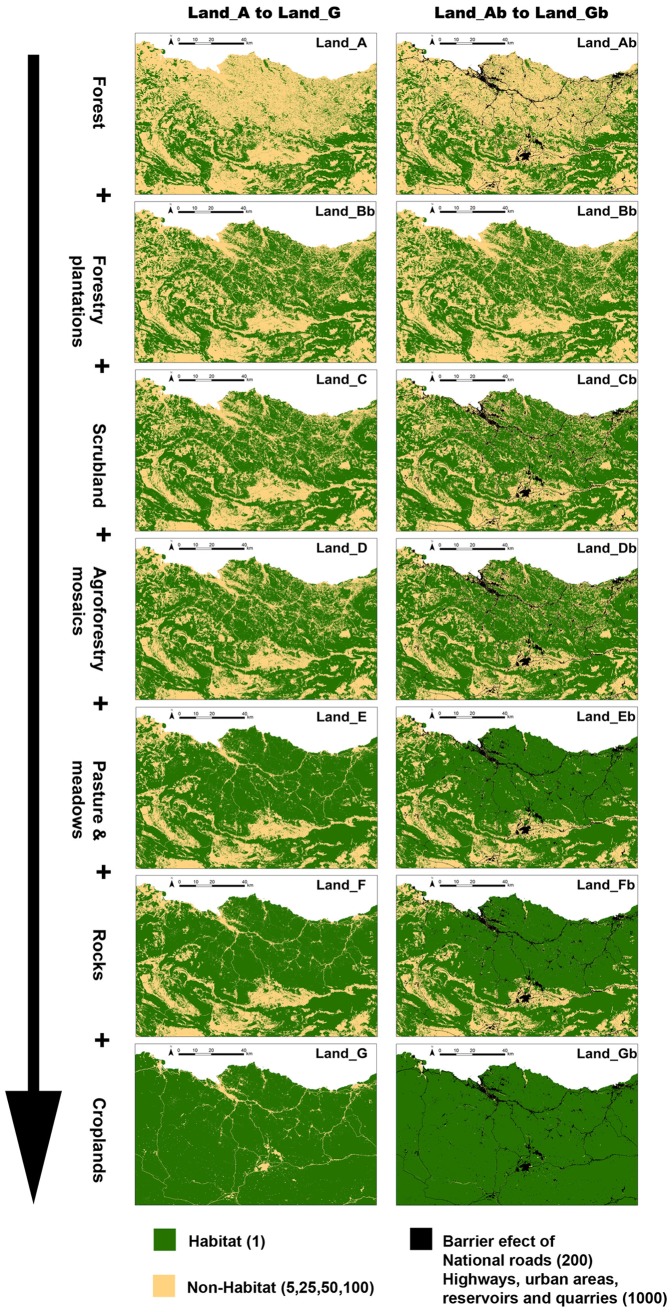
Binary landscape resistance maps on a gradient from greater to lesser preference of the focal species in relation to forest environment. Binary landscape resistance maps, on a gradient from greater to lesser preference of the focal species in relation to forest environment (Land_A to Land_G). Green-coloured cells represent “Habitat” (resistance value 1) and yellow-coloured cells “Non-Habitat” (resistance values evaluated, 5, 25, 50,100). Models Land_Ab to Land_Gb additionally include black-coloured cells representing the barrier effect of national roads (resistance value 200), highways, urban areas, reservoirs and quarries (resistance value 1000).

**2) Binary Landscape resistance maps:** Our second set of hypotheses propose that some land uses promote genetic connectivity for forest dependant species, such as the pine marten, that specialize in such habitats [47], [48], while others resist gene flow. Thus, different binary resistance maps were developed to evaluate the specific land uses which were favourable and unfavourable to the dispersal movements of the martens (i.e. habitat *vs* non-habitat-model). As pine martens are believed to need a minimum woodland area to survive (ca. 2km^2^) [47] and tend to avoid treeless areas [48], [49], [51], we expected to find a positive effect of closed-canopy forest habitats and negative effect of open areas and human transformed landscapes on gene flow. Thus, the different binary resistance maps created (Land_A, Land_B, Land_C, Land_D, Land_E, Land_F and Land_G) covered a gradient from greater to lesser preference of the focal species for forest environments, ranging from strictly forest land (Land_A) up to and including open spaces (Land_G) (Table 1, Fig. 2). Therefore, we classified land use data as habitat *vs*. non-habitat and parameterized the models according to a range of plausible resistance values. As there is not a general rule for the assignment of resistance values to non-habitat, we explored 4 different resistance values (5, 25, 50, 100) of non-habitat relative to habitat to verify if it could affect the detectability of landscape genetic relationships, as it has been previously shown in both empirical [24] and simulation [84] studies. In this way, preferential land uses for dispersal were assigned a value of 1 (i.e. Habitat), while non-favourable to dispersal habitat (i.e. non-habitat) sites were assigned a value of 5, 25, 50 or 100, depending on the scenario.

For each of the resistance maps described above, we tested the effect of potential anthropogenic barriers, including national roads (resistance 200), highways, urban areas, reservoirs and quarries (resistance 1000) (Land_Ab to Land_Gb resistance maps; Table 1), using the same resistance values of the ecological network resistance map (see below), for comparative purposes [41]. As each resistance map (Land_A to Land_G; Land_Ab to Land_Gb) was explored for 4 different resistance values, we evaluated 56 different binary resistance maps (i.e. without barrier effect: Land_A_x_ to Land_G_x_; With barrier effect: Land_Ab_x_ to Land_Gb_x_; where X corresponds to the 4 evaluated resistances values per model: 5, 25, 50, and 100) (Table 1).

**3) Ecological network resistance map:** We evaluated the resistance map previously utilized in the design of ecological corridors linking forest Natura 2000 areas of the Basque Country [41]. The resistance map was based on the assignment of different resistance levels to each land use and parameterized through bibliographical review and expert opinion [41]. The resistance surface outlined in [41] was updated with the new available spatial data regarding land uses in the study area [59], [60] (Table 1). Raster breaks in linear barrier elements were avoided by the reinforcement of the size of national roads and highways [14]. Sections of highways which run through viaducts or tunnels were assigned the resistance value corresponding to the land use of the surrounding area. Additionally, a second resistance map was used, with a view to testing the effect of noticeably decreasing the resistance value attributed to the potential barrier effects of national roads, highways, urban areas, reservoirs and quarries (ENnb map) (Table 1).

The raw ascii file of the EN is included in File S1. Following the resistance values outlined in Table 1, all the 59 resistance maps evaluated can be produced from the raw ascii EN resistance map (File S1).

The effective and Euclidean distances between each pair of individuals were calculated with PATHMATRIX 1.1 [15]. Pair-wise effective distances between individuals were calculated as the accumulated cost through the least cost paths (LCP) throughout each resistance surface [14], [15] (Fig. 1).

We proposed 59 alternative landscape models: 1) 56 binary landscape resistance maps; 2) two complex resistance maps based on the resistance surfaces used to develop the regional ecological network (EN and ENnb) (Table 1), and 3) the null model of Isolation by Distance.

### Relationship between genetic and geographical distances within a reciprocal causal modeling framework

#### Mantel correlations between genetic distance and alternative resistance hypotheses

The pairwise genetic distances matrix (Rousset's a_r_) was correlated with different matrices of geographical and (cost) distances encompassing a total number of 5151 pairwise comparisons, including: i) Euclidean distance, to determine whether the patterns of differentiation follow an isolation by distance pattern (null hypothesis) and ii) the effective distances calculated for each of the 58 resistance maps, to infer landscape structure effects on gene flow. The correlation between distance matrixes was calculated by means of the Mantel test [85] and partial Mantel tests [86] as implemented in the ECODIST package [86] in R version 2.7 (R Development Core Team 2008) with 10,000 permutations. Given the potential sensitivity of Mantel tests to non-linear relationships between genetic and cost-distances [87], we compared results between two sets of analyses, one log transforming the effective and Euclidean distances, and one using the original untransformed cost-distance matrices.

#### Factorial hypothesis cube randomization: Evaluation of the unimodality of support across landscape models

When hypotheses are constructed across a quantitative range of values for a parameter, it is possible to evaluate the degree to which the analysis indicates a unimodal peak of support for a global best model [30]. The degree of unimodality of model support in a factorial hypothesis cube is one measure of the reliability of model results [13]. This is done by computing the differences in support (in our case partial Mantel r values) among all neighbouring cells (i.e. different models) in the hypothesis cube and comparing the sum of those differences to the distribution of the sum of differences from a large number of randomizations of the hypothesis cube (e.g. [13]). We evaluated the unimodality of support across the 56 binary resistance hypotheses (i.e. without barrier effect: Land_Ax to Land_Gx; With barrier effect: Land_Abx to Land_Gbx; where X corresponds to the 4 evaluated resistances values per model: 5, 25, 50, and 100) for the transformed and untransformed analyses using the randomization procedure introduced by Cushman et al. [13], in which the order of hypotheses in the hypothesis cube is randomized a large number of times and each time the difference in partial Mantel r (partialling out distance) is calculated between neighboring hypotheses in the cube. The sum of squared neighbor distances from the actual hypothesis cube (Actual Sum Differences, ASD) is then compared to the distribution of squared neighbor distances in the randomized hypothesis cubes (Mean Sum Randomized Differences, MSRD). We conducted this analysis with 1,000,000 randomizations of the hypothesis cube for both the untransformed and transformed analysis. If no randomizations produce a sum of squared neighbor distances as small as observed, it is strong evidence that the analysis has shown a strong peak of support (i.e. unimodal support).

#### Original causal modeling

In addition to the reciprocal causal modeling approach [84] (see below), we conducted the original causal modeling [30] as a comparative framework, in which the 58 alternative landscape resistance models are tested against the null model of isolation by distance (IBD) as described in Cushman et al. [30]. There were 3 sets of diagnostic Mantel and partial Mantel tests to complete the causal modeling. These included: (i) simple Mantel tests between genetic distance and landscape resistances; (ii) partial Mantel tests between genetic distance and landscape cost distances, partialling out the effects of Euclidean distance; (iii) partial Mantel tests between genetic distances and Euclidean distance, partialling out the effects of landscape resistance. To infer an effect of a landscape resistance scenario on dispersal, we expected (i) and (ii) to be significant, and we expected (iii) to be negative or non-significant if that scenario ‘correctly’ explained population connectivity in our study population [30].

#### Reciprocal causal modeling

We used (partial) Mantel tests in a reciprocal causal modeling framework [84] to analyse the influence of landscape structure on gene flow and to determine the extent to which possible landscape resistance models (i.e. resistance maps) explained the spatial pattern of genetic distance between individuals. Cushman and Languth [88] found that the inherent high correlation among alternative resistance models results in a high risk of spurious correlations using simple Mantel tests. Several refinements, including causal modeling [30], have been developed to reduce the risk of affirming spurious correlations and to assist model selection. However, Cushman et al. [84] showed these still suffer from elevated Type I error rates. Consequently, Cushman et al. [84] proposed “reciprocal causal modeling” which they showed greatly lessens Type I error rates in landscape genetic analysis [89]. In reciprocal causal modeling, each alternative resistance hypothesis is tested against all others with partial Mantel tests. A matrix of relative support is calculated by taking the difference between a) the partial Mantel r of each candidate model partialling out each alternative model, and b) the partial Mantel test of the alternative model partialling out the candidate model [84]. A fully supported hypothesis will have positive values of this difference with all alternative models, and no alternative models will have positive values compared to the supported model.

## Results

### Non-invasive sample collection and species identification

Out of 733 faecal samples collected from the entire study area, 141 were discarded because they were not fresh or because they presumably belong to the same individual (samples separated by <1km). 494 out of 592 analyzed samples were classified as *Martes* sp. (*M. martes* and *M. foina*) based on genetic species identification results. Thus, unequivocal species identification was possible in 83.45% of the samples. We effectively identified 232 faecal samples as stone marten and 262 as pine marten. Additionally, we obtained 57 tissue samples from road-killed pine martens.

Out of 262 faecal samples identified as pine marten, 108 were not included to the microsatellite genotyping procedure. These samples correspond to the sampling period from 2004–2005, which was used for a first distribution assessment of sympatric martens in the study area and were not potentially fresh enough for microsatellite analysis. Thus, 213 pine marten samples (154 faecal samples and 59 tissue samples) were used for microsatellite genotyping.

### Individual identification, genotype checking and genetic diversity

The first quality-screening test, based on 4 replicates of four loci, was not passed by 73 non-invasive samples (47.40%), which were immediately discarded. The remaining 81 samples (52.59%) were amplified at the other 11 loci. After multiple-tubes genotyping, 27 samples from this sub-set (17.53% from the total analyzed samples) were then discarded because they showed <50% PCR success, or because of high failure rates. Full multilocus microsatellite genotypes were obtained for the remaining 54 samples (66.67% from the samples that passed the screening and 35.06% from the total samples analyzed) all showing reliability score> 0.95 [67].

The observed average error rates across loci were: ADO = 0.188 and FA = 0.017. PID analysis showed that the set of 15 loci would produce an identical genotype with a probability of 1.69×10^−10^, and with a probability of 4.45×10^−5^ for a full-sib, suggesting no “shadow effect” (i.e. all the genotypes identify distinct individuals; [90], and that matching genotypes were recaptures of the same individual).

After a regrouping procedure, we identified 42 individual genotypes from faecal samples. All of the 59 tissue samples were correctly genotyped at 15 loci and all provided new individuals. In total we identified 113 genotypes that corresponded with 101 different individuals. The number of times each individual was detected in the survey varied from 1 to 3, with a total number of 12 re-samplings. Complete genetic profiles and the geographic coordinates for the 101 pine marten individuals are included in Table S2.

The average observed (HO) and expected (HE) heterozygosity values were 0.53 and 0.58, respectively (Table S1). All 15 loci were variable with total numbers of alleles ranging between 3 and 8 per locus. The overall pine marten dataset showed a significant deficit of heterozygotes as compared to Hardy-Weinberg expectations (p <0.001). Despite the broad scale of sampling, the majority of loci were in Hardy-Weinberg proportions (13 out of 15). Only loci Mp0188 and Lut-435 were out of Hardy-Weinberg proportions (Table S1). These results suggest signs of a Wahlund effect, due to the existence of an isolation by distance (Euclidean or effective) pattern in the study area. Linkage disequilibrium was not apparent for any pair of loci after performing Bonferroni corrections.

### Correlation between genetic and effective distances

#### Factorial hypothesis cube randomization

**Figure 3:**
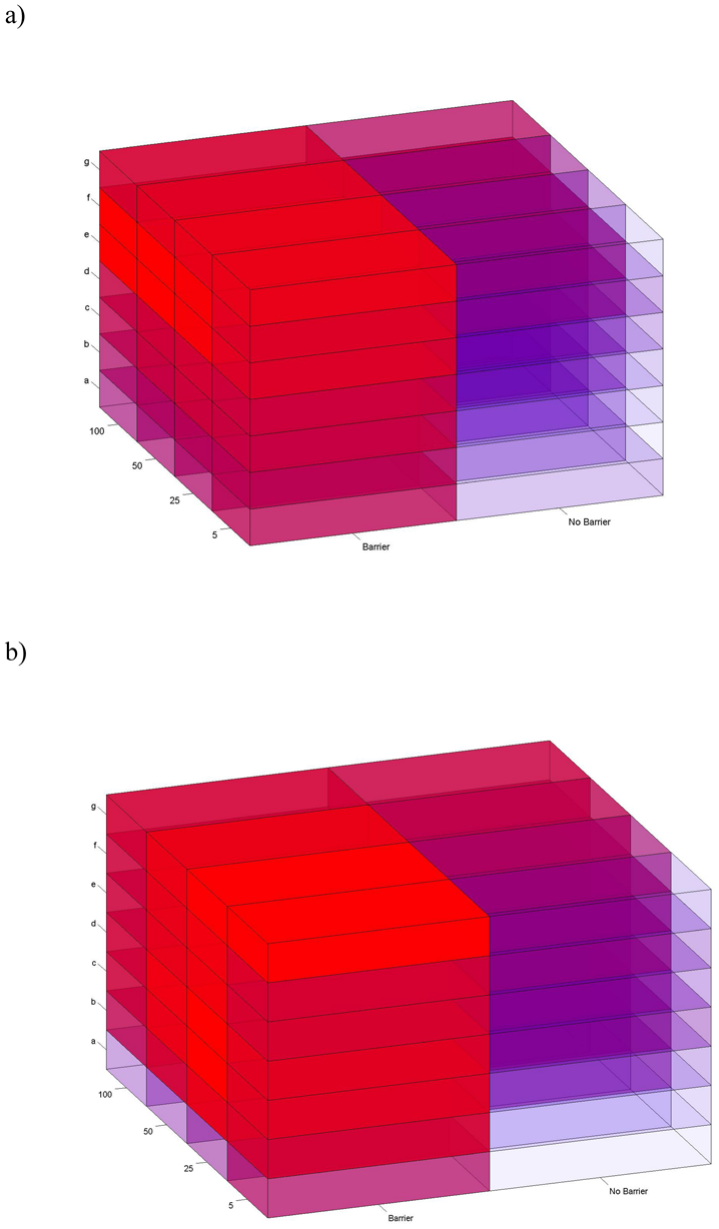
Factorial hypothesis cube randomization. Visualization of the 56 binary landscape-resistance hypotheses after the effects of geographical distance are partialed out on the a) log transformed and b) untransformed cost distances. The cubes each represent one of the 56 binary landscape-resistance models. The cubes are colored in a gradient from blue to red, with red being the most supported models based on the partial Mantel r value. The Mantel r values corresponding to each cube are found in Table 2 and Table S3 for the log transformed and the untransformed matrices, respectively.

We evaluated the unimodality of support across the 56 binary resistance maps for the log transformed and untransformed data to determine which form of the data should be used for subsequent analyses. After the effects of distance are partialled out, ranking the models by partial Mantel r value provides a means to determine which hypotheses have the greatest support and to identify the most related model to the genetic structure (Table 2, Fig. 3). According to the results outlined in Figure 3, there is a more coherent, unimodal pattern of support in the transformed analysis than the untransformed analysis. Additionally, factorial randomization of the hypothesis cube, in both the transformed and untransformed analyses, no instance of 1,000,000 randomizations produced a sum of squared differences between neighboring hypotheses (MSRD) as small as the actual sum of squared differences (ASD) in partial Mantel r values (Table 3), indicating very high unimodality in both forms of analysis. However, the transformed analysis had higher total support for optimal unimodal support of the best hypothesis as indicated by the larger number of standard errors of MSRD between neighboring hypotheses across the 1,000,000 randomizations (Table 3). Accordingly, all subsequent analyses are restricted to the log transformed resistance distances. As indicated by the hypothesis cube (Fig. 3), the different resistance values evaluated (5, 25, 50, 100) slightly modified the (partial) Mantel correlation results obtained for each model for both the log transformed (Table 2; Fig. S1 and Fig. S2) and the untransformed distances (Table S3; Fig. S3 and Fig. S4), but overall a consistent pattern was obtained.

**Table 2:** Results of causal modeling of landscape resistance on genetic distance in European pine marten according to Mantel and partial mantel tests.

	Model/Resistance Values	1) Simple mantel		Rank	2) Partial mantel test		Rank	3) Partial mantel		CMS?		1) Simple mantel test		Rank	2) Partial mantel test		Rank	3) Partial mantel		CMS?
		G*L	G*L		G*L|Dis	G*L|Dis		G*Dis|L	G*Dis|L			G*L	G*L		G*L|Dis	G*L|Dis		G*Dis|L	G*Dis|L	
		R	p		R	p		R	p			R	p		R	*p*		R	p	
**Binary Landscape Resistance Models (Land_A to Land G)**	**Land_A** *Forest*										**Binary Landscape Resistance Models (Land_Ab to Land_Gb)**	**Land_Ab**								
	5	*0.221*	*0.0001*	49	0.057	0.202	54	*−0.007*	*0.877*	**N**		*0.243*	*0.0001*	28	*0.126*	*0.0005*	26	*−0.045*	*0.256*	**Y**
	25	*0.219*	*0.0001*	53	0.066	0.215	51	*0.047*	*0.317*	**N**		*0.243*	*0.0001*	29	*0.117*	*0.0138*	32	*0.008*	*0.861*	**Y**
	50	*0.217*	*0.0001*	56	0.074	0.170	49	*0.066*	*0.156*	**N**		*0.238*	*0.0001*	34	*0.112*	*0.0279*	36	*0.038*	*0.421*	**Y**
	100	*0.211*	*0.0001*	58	0.080	0.138	46	*0.089*	*0.053*	**N**		*0.229*	*0.0001*	42	*0.107*	*0.0445*	38	*0.067*	*0.159*	**Y**
	*Mean (*±*SE)*	**0.2169 (±0.0044)**			**0.0692 (±0.0099)**							**0.2381 (±0.0063)**			**0.1152 (±0.0078)**					
	**Land_B** *Forest + Forestry plantations)*											**Land_Bb**								
	5	*0.216*	*0.0001*	57	0.027	0.463	58	*0.011*	0.764	**N**		*0.242*	*0.0001*	30	*0.125*	*0.0001*	27	*−0.050*	*0.161*	**Y**
	25	*0.224*	*0.0001*	47	0.068	0.134	50	*0.009*	0.838	**N**		*0.245*	*0.0001*	23	*0.124*	*0.0022*	28	*−0.028*	*0.511*	**Y**
	50	*0.227*	*0.0001*	46	0.082	0.085	45	*0.024*	0.612	**N**		*0.245*	*0.0001*	25	*0.121*	*0.0083*	29	*−0.004*	*0.938*	**Y**
	100	*0.232*	*0.0001*	40	0.099	0.051	41	*0.040*	0.380	**N**		*0.241*	*0.0001*	32	*0.117*	*0.0195*	31	*0.028*	*0.539*	**Y**
	*Mean (*±*SE)*	**0.2247 (±0.0069)**			**0.0689 (±0.0309)**							**0.2432 (±0.0018)**			**0.1220 (±0.0037)**					
	**Land_C** *Forest + forestry plantations + Scrublands*											**Land_Cb**								
	5	*0.218*	*0.0001*	54	0.041	0.128	56	*−0.006*	0.863	**N**		*0.245*	*0.0001*	24	*0.135*	*0.0002*	21	*−0.059*	*0.105*	**Y**
	25	*0.228*	*0.0001*	45	*0.080*	*0.043*	47	*−0.009*	0.840	**Y**		*0.250*	*0.0001*	14	*0.140*	*0.0005*	18	*−0.045*	*0.305*	**Y**
	50	*0.232*	*0.0001*	41	*0.091*	*0.033*	43	*0.008*	0.868	**Y**		*0.251*	*0.0001*	11	*0.137*	*0.0022*	20	*−0.024*	*0.587*	**Y**
	100	*0.239*	*0.0001*	33	*0.111*	*0.018*	37	*0.021*	0.648	**Y**		*0.248*	*0.0001*	17	*0.129*	*0.0085*	25	*0.009*	*0.859*	**Y**
	*Mean (*±*SE)*	**0.2291 (±0.0088)**			**0.0806 (±0.0292)**							**0.2487 (±0.0028)**			**0.1349 (±0.0045)**					
	**Land_D** *Forest + forestry plantations + scrublands + agroforestry mosaics*											**Land_Db**								
	5	*0.220*	*0.0001*	52	0.052	0.161	55	*−0.019*	0.608	**N**		*0.246*	*0.0001*	22	*0.137*	*0.0001*	19	*−0.062*	*0.090*	**Y**
	25	*0.229*	*0.0001*	44	*0.083*	*0.041*	44	*−0.012*	0.801	**Y**		*0.251*	*0.0001*	12	*0.142*	*0.0006*	15	*−0.047*	*0.280*	**Y**
	50	*0.235*	*0.0001*	37	*0.100*	*0.038*	40	*−0.001*	0.981	**Y**		*0.253*	*0.0001*	8	*0.140*	*0.0024*	17	*−0.027*	*0.564*	**Y**
	100	*0.242*	*0.0001*	31	*0.116*	*0.026*	33	*0.017*	0.721	**Y**		*0.251*	*0.0001*	13	*0.134*	*0.0064*	22	*0.005*	*0.915*	**Y**
	*Mean (*±*SE)*	**0.2313 (±0.0095)**			**0.0877 (±0.0272)**							***0.2501 (±0.0031)***			***0.1382 (±0.0036)***					
	**Land_E** *Forest + forestry plantations + scrublands + agroforestry mosaics + pastures*											**Land_Eb**								
	5	*0.220*	*0.0001*	51	*0.062*	*0.049*	53	*−0.035*	0.265	**Y**		0.248	*0.0001*	19	*0.149*	*0.0001*	13	*−0.078*	*0.016*	**Y**
	25	*0.234*	*0.0001*	39	*0.113*	*0.009*	35	*−0.061*	0.132	**Y**		0.255	*0.0001*	7	*0.167*	*0.0001*	6	*−0.089*	*0.015*	**Y**
	50	*0.243*	*0.0001*	27	*0.133*	*0.004*	24	*−0.064*	0.142	**Y**		*0.259*	*0.0001*	**4**	*0.171*	*0.0001*	**2**	*−0.086*	*0.032*	**Y**
	100	*0.253*	*0.0001*	10	*0.150*	*0.002*	11	*−0.061*	0.176	**Y**		0.261	*0.0001*	**2**	*0.170*	*0.0002*	**4**	*−0.074*	*0.097*	**Y**
	*Mean (*±*SE)*	**0.2373 (±0.0138)**			**0.1146 (±0.0380)**							**0.2556 (±0.0060)**			**0.1638 (±0.0103)**					
	**Land_F** *Forest + forestry plantations + scrublands + agroforestry mosaics + pastures + rocky areas*											**Land_Fb**								
	5	*0.220*	*0.0001*	50	*0.062*	*0.044*	52	−0.035	0.266	**Y**		*0.248*	*0.0001*	20	*0.149*	*0.0001*	12	*−0.078*	*0.016*	**Y**
	25	*0.234*	*0.0001*	38	*0.114*	*0.008*	34	*−0.062*	0.134	**Y**		*0.255*	*0.0001*	6	*0.167*	*0.0001*	**5**	*−0.089*	*0.014*	**Y**
	50	*0.243*	*0.0001*	26	*0.134*	*0.004*	23	*−0.064*	0.140	**Y**		*0.259*	*0.0001*	**3**	*0.171*	*0.0001*	**1**	*−0.086*	*0.028*	**Y**
	100	*0.253*	*0.0001*	9	*0.150*	*0.003*	10	*−0.061*	0.184	**Y**		*0.262*	*0.0001*	**1**	*0.170*	*0.0002*	**3**	*−0.074*	*0.088*	**Y**
	*Mean (*±*SE)*	**0.2374 (±0.0138)**			**0.1150 (±0.0381)**							**0.2557 (±0.0060)**			**0.1640 (±0.0104)**					
	**Land__G** *Forest + forestry plantations + scrublands + agroforestry mosaics + pastures + rocky areas + croplands*											**Land_Gb**								
	5	*0.217*	*0.0001*	55	*0.041*	*0.027*	57	−0.018	0.351	**Y**		*0.249*	*0.0001*	16	*0.157*	*0.0001*	7	*−0.089*	*0.007*	**Y**
	25	*0.223*	*0.0001*	48	*0.074*	*0.002*	48	−0.039	0.125	**Y**		*0.249*	*0.0001*	15	*0.157*	*0.0001*	8	*−0.087*	*0.009*	**Y**
	50	*0.229*	*0.0001*	43	*0.096*	*0.000*	42	−0.051	0.077	**Y**		*0.248*	*0.0001*	18	*0.152*	*0.0001*	9	*−0.081*	*0.017*	**Y**
	100	*0.237*	*0.0001*	36	*0.120*	*0.000*	30	−0.063	0.043	**Y**		*0.246*	*0.0001*	21	*0.142*	*0.0001*	16	*−0.071*	*0.036*	**Y**
	*Mean (*±*SE)*	**0.2263 (±0.0082)**			**0.0827 (±0.0338)**							**0.2481 (±0.0014)**			**0.1519 (±0.0069)**					
	**ENnb**											**EN**								
		*0.237*	*0.0001*	35	*0.103*	*0.032*	39	*−0.003*	0.947	**Y**		*0.256*	*0.000*	***5***	*0.146*	*0.0018*	14	*−0.030*	*0.519*	**Y**

Model definitions according to Table 1. There are 3 Mantel tests comprising causal modeling: (1) G*L—simple Mantel test between the candidate model and genetic distance; (2) G*L|Dis—partial Mantel test between the candidate model and genetic distance, partialling out Euclidean distance; (3) G*D|L—partial Mantel test between the Euclidean and genetic distance, partialling out the candidate model. For a candidate model to be supported tests (1) and (2) must be significant, while test (3) must be negative or non-significant. Mantel tests meeting each criterion are italicized. Ranking of each model according to Mantel and partial Mantel r values is included. CMS? Indicates if the model is supported within the causal modeling framework (Y) or not (N).

**Table 3:** Factorial randomization of the hypothesis cube.

	Untransformed	Transformed
Rank	1	1
Actual Sum Differences (ASD)	19.6325	17.81
Mean Sum Randomized Differences (MSRD)	26.4738	24.17
SD error from MSRD	6.59E+04	6.97E+04

#### Simple Mantel correlations between genetic differentiation and alternative landscape models

**Figure 4:**
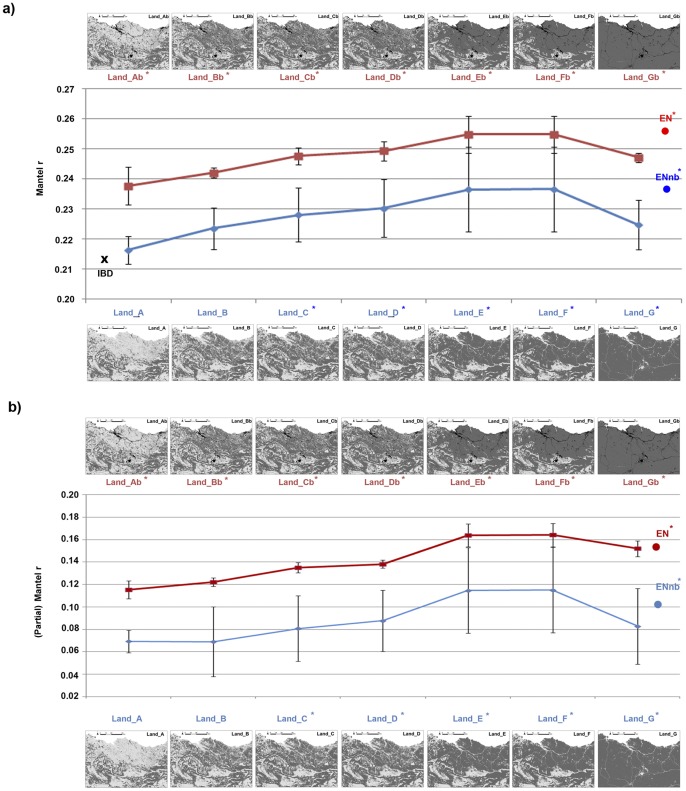
Mantel r results for the different landscape resistance maps evaluated (log transformed). **a**) Pearson correlation coefficients (Mantel r) between genetic distance and effective distance (log transformed) and **b**) Pearson correlation coefficients (Partial Mantel r) between genetic distance and effective distance (log transformed) after factoring out the effect of the Euclidean distance in the different landscape resistance maps examined. Models marked with an asterisk correspond to the models supported within the causal modeling framework [30].

*1) Isolation by distance:* A significant positive correlation was obtained between the genetic distances and Euclidean distances (r = 0.214; p <0.0001), bearing clear witness to the existence of a pattern of isolation by distance (IBD) (Table 2, Fig. 3). However, when the models were ranked based on Mantel r, all the landscape models performed better than the null model (Table 2, Fig. 4).

*2) Binary resistance maps:* All of the simple Mantel tests were significant when analyzed in log-transformed form (Table 2). The correlation between genetic and effective distance gradually increased on including, in addition to natural forest (Land_A), forestry plantations (Land_B), scrublands (Land_C), agroforestry mosaics (Land_D), and pastures and meadows (Land_E) as environments favouring dispersal (Table 2, Fig. 4). This correlation did not change on including rocky areas as dispersal environments (Land_F), while it decreased on including cultivated land (Land_G). The same pattern was obtained with models which specifically increase the cost value of the main barrier features (national roads, highways, urban areas, reservoirs and quarries; Land_Ab to Land_Gb resistance maps), but with an increase of Mantel r values with respect to Land_A to Land_G models (Table 2, Fig. 4), indicating that including barrier effects due to linear features improves the resistance model. The correlation reached its maximum value on including barrier effects in resistance maps Land_E and Land_F (i.e. resistance maps Land_Eb and Land_Fb; r = 0.256±0.0060; p <0.0001) (Table 2, Fig. 4). The different resistance values (5, 25, 50, 100) slightly modified the correlation results obtained for each model (see Figure S2 and S4 for further details), with the highest correlations for Land_Fb100 (Table 2, Table S3; Fig. 4).

*3) Ecological network resistance map:* The effective distances calculated on the basis of the EN map were positively correlated with genetic distances and explained a slightly higher proportion of the observed genetic variance than the Euclidean distances (EN, r = 0.256; p <0.0001; Table 2, Fig. 4). The degree of correlation when using the ENnb map was less than that obtained with EN, though still greater than that obtained using Euclidean distance (r = 0.237; p <0.0001; Table 2, Fig. 4). However, the original model used in the design of the ecological network (EN), which included a higher barrier effect for national roads, highways, urban areas, reservoirs and quarries was better supported than the alternative model (ENnb).

#### Partial Mantel correlations between genetic differentiation and alternative resistance hypotheses

We found significant effects of nearly all of the landscape resistance models (46 out of 52), as the relationship between genetic distance and effective distance was always significant when Euclidean distance was factored out of the relationship (p <0.05) (Table 2; Table S3).

*1) Binary landscape resistance maps:* The correlation values after factoring out the effect of Euclidean distance showed the same pattern of increase of that obtained by means of a simple Mantel test, with Land_Fb50 showing the highest partial Mantel r correlation (Table 2; Figure 4 and Fig. S2). However, Land_A_x_, Land_B_x_, Land_C5 and Land_D5 were not significant when the Euclidean distance was partialled out. Factorial support cubes indicate a clear unimodal peak of support in models Land_Fb50, Land_Eb50, Land_Fb100, Land_Eb100 (Figure 3) that are ranked from first to fourth according to partial r values. Similarly, these models have the highest simple Mantel r values of all of the evaluated models (Table 2; Figure 4). These results suggest that there is a strong peak of support for Land_Fb50 with a clear similarity with Land_Eb50, Land_Fb100 and Land_Eb100. Thus, the best supported models were associated with minimum resistance to movement on forest, forestry plantations, scrublands, agroforestry mosaics and pastures habitats and clear support for the barrier effect.

*2) Ecological network resistance map:* Both EN and ENnb models appeared better supported than the null model of IBD as this latter retained a significant positive relationship with a_r_-based genetic distance after factoring out the effects of Euclidean distance, but less supported than the top resistance models (Land_Eb50, Land_Fb100, Land_Eb100; Table 2).

#### Original and reciprocal causal modeling

Using the original form of causal modeling approach [30] we found that all binary resistance hypotheses except Land_Ax, Land_Bx, Land_C5 and Land_D5 were supported (Table 2).

**Figure 5:**
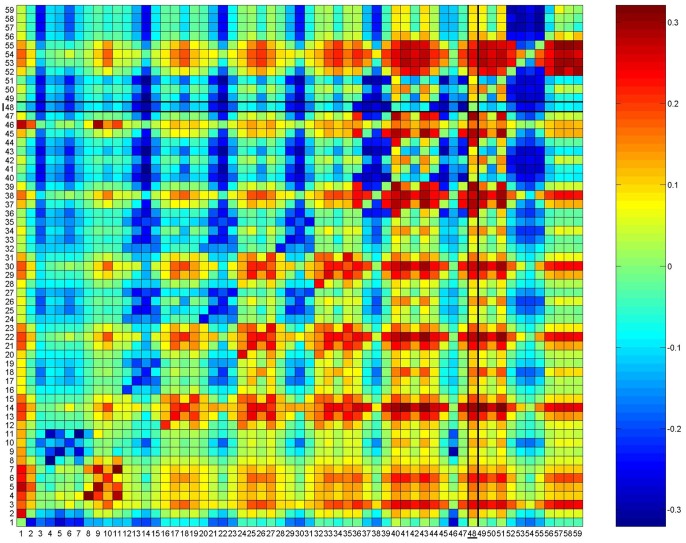
Reciprocal causal modeling results. Results of reciprocal causal modeling on the log transformed cost distances. A single resistance model (Model 48-Land_Fb100) is supported in analysis of the transformed cost distances. Columns indicate focal models, and rows indicate alternative models. The color gradient from blue to red indicates support for the focal model independent of the alternative model (e.g. focal model | alternative model – alternative model | focal model is positive). A fully supported model would have all positive values in the vertical dimension (e.g. that model is supported independently of all other models), and all negative values in the horizontal dimension (no other model is supported independently of the focal model). Model number and associated resistance map: 1 - EN, 2 - ENnb, 3 - Geo_dist, 4 - Land_A100, 5 - Land_A25. 6- Land_A5. 7 - Land_A50, 8 - Land_Ab100, 9 - Land_Ab25, 10 - Land_Ab5. 11 - Land_Ab50. 12 - Land_B100, 13 - Land_B25, 14 - Land_B5, 15 - Land_B50, 16 - Land_Bb100, 17 - Land_Bb25, 18 - Land_Bb5, 19 - Land_Bb50, 20 - Land_C100, 21 - Land_C25, 22- Land_C5, 23 - Land_C50, 24- Land_Cb100, 25 - Land_Cb25, 26 - Land_Cb5, 27 - Land_Cb50, 28 - Land_D100, 29 - Land_D25, 30 - Land_D5, 31 - Land_D50, 32 - Land_Db100, 33 - Land_Db25, 34 - Land_Db5, 35 - Land_Db50, 36 - Land_E100, 37 - Land_E25, 38 - Land_E5, 39 - Land_E50, 40 - Land_Eb100, 41 - Land_Eb25, 42 - Land_Eb5, 43 - Land_Eb50, 44 - Land_F100, 45 - Land_F25, 46 - Land_F5, 47 - Land_F50, 48 - Land_Fb100, 49 - Land_Fb25, 50 - Land_Fb5, 51 - Land_Fb50, 52 - Land_G100, 53 - Land_G25, 54 - Land_G5, 55 - Land_G50, 56 - Land_Gb100, 57 - Land_Gb25, 58 - Land_Gb5, 59 - Land_Gb50.

Using the novel Cushman et al. [84] method of "reciprocal causal modeling", only one resistance model out of the 59 candidate models was fully supported. The single supported model is model number 48, Land_Fb100. The reciprocal causal modeling method shows that the indexes of relative support of this model [i.e. calculated by taking the difference between 1) the partial Mantel r value of the candidate model partialling out each alternative model and 2) the partial Mantel r of the alternative model partialling out the candidate model (reciprocal partial Mantel test)] are all positive (Fig. 5). In addition to model Land_Fb100, several others are nearly perfectly supported. Land_Eb100 is supported independently of all but model Land_Fb100. Similarly, Land_Fb50 is supported independently of all except Land_Fb100 and Land_Eb100. Model Land_Eb50 is supported independently of all but Land_Fb100, Land_Fb50, and Land_Eb100. This verifies the peak of support seen in the hypothesis cube, with highest support for Land_Fb100, followed by Land_Eb100, Land_Fb50, and Land_Eb50 [i.e. models that included in addition to natural forest, forestry plantations, scrublands, agroforestry mosaics, pastures and meadows (Land Eb) and rocky areas (Land_Fb) as environments favouring dispersal and importance effect of roads as potential barriers to gene flow]. Importantly, the Ecological Network model EN was supported independently of all other models except Land_Fb100, Land_Eb100, Land_Fb50 and Land_Eb50, indicating that it is a highly effective surrogate for landscape resistance to pine marten gene flow.

## Discussion

Recent studies suggested that individual-based landscape genetic analysis using partial Mantel tests in a causal modeling framework have high power to correctly identify landscape resistance as a driving process and reject spurious correlations with isolation by distance [84], [88]. Cushman et al. [84] showed that the reciprocal causal modeling method we employed here substantially reduces the frequency of Type I errors. In this regards, Castillo et al. [91] recently showed that simulations support the reciprocal causal modeling with partial Mantel tests approach as an effective means to identifying the relationship between gene flow and landscape variables. Although there has been recent controversy over the use of Mantel tests in landscape genetics [92]–[94], a preferable alternative has yet to be identified that does not also suffer drawbacks [84]. There is no one-size- fits-all approach, and the most appropriate methodology will depend on the research question and landscape under investigation [92]. We use here the more robust modeling framework, proposed by Cushman et al. [84], that is based on the relative support of each candidate model and includes a reciprocal causal modeling step in the model optimization process. Moreover, we used the original causal modeling approach [30], factorial hypothesis cube randomization and ranking by simple Mantel test values as a comparative framework to further explore the performance of these several approaches.

Our results clearly indicate that a standard isolation-by-distance model is not sufficient to explain the observed genetic pattern, and including landscape variables through different resistance maps significantly improves the prediction of the target species gene flow. One model was supported in the transformed analysis using reciprocal causal modeling. This model, Land_Fb100, indicates that pine marten gene flow in northern Spain is facilitated by forests, forestry plantations, scrubland, agroforestry mosaics and pastures and meadows, and that crops have roughly 100 times higher resistance than optimal habitat. Further, this uniquely supported model indicates that anthropogenic barriers, such as national roads, highways, urban areas, reservoirs and quarries and wetlands likely pose much greater resistance to marten gene flow. This suggests that the population connectivity of pine martens in the study area may be vulnerable to habitat loss and fragmentation processes, due to the presence of anthropogenic barriers as has been previously suggested in other forest-dependant species [25], [32], [95].

The reciprocal causal modeling approach [84] clearly improved discrimination among the competing models, from only one fully supported model (i.e. Land_Fb100) versus more than 80% of models supported equivocally by the original form of causal modeling [30]. Indeed, recent studies showed that the novel reciprocal causal modeling approach is a strong improvement over other methods [91], [96]. Even thought reciprocal causal modeling has a greater discrimination to detect the top model, the Cushman et al. [30] method (i.e. causal modeling + model rank + hypothesis cube) reached nearly identical conclusions. We found that ranking models based on simple or partial Mantel r gave consistent support. Likewise, our evaluation of unimodality of support in the hypothesis cube (e.g. [13], [24], [30]) verified the same peak of support. Reciprocal causal modeling resolved the Type I error problem that we saw in our results from the original form of causal modeling (evaluating support relative to isolation by distance), and was consistent with the peak of support seen in the factorial hypothesis cube. Thus, both forms of causal modeling are complementary and provided independent support to the obtained results, as did evaluating unimodality of support in the hypothesis cube.

In landscape genetics a large number of pairwise genetic relatedness measures have been applied to infer effects of landscape structure on gene flow [12], [97], with Rousset's a_r_ or â [75] being one of the most widely used measures (e.g. [79], [80], [98], [99]). Watts et al. [100] proposed a differentiation statistic (ê) that seems to improve Rousset's â performance for populations with large neighborhood-size (Dσ2) values (i.e. weak IBD pattern). However, differences in statistical performance usually highly depend on the data set used and the sampling scheme as well as how well the data set meets the underlying model assumptions [77]. Thus, and even if the results among different genetic distance measures have generally agreed [24], [29], [30], [101] further studies based on empirical analyses of genetic patterns and simulation modeling are needed to properly evaluate the potential effect of different genetic distance estimator on disentangling landscape effects on gene flow.

### Influence of land uses on gene flow: insights into pine marten ecology

The most consistent marten-habitat relation appears to be a general association with forest habitats, and avoidance of open, non-forested habitats [48], [51], [52]. Thus, the marten's unwillingness to cross open habitats may restrict the species' ability to disperse and colonise new forested areas [51], [55]. Ruiz-González et al. [52] found that pine marten occurrence in the study area is highly dependent on the presence of forest and consequently sensitive to forest fragmentation as has been previously suggested in other studies across Europe [49], [51]. Nevertheless, the presence of forest habitats is not the only factor which explains pine marten gene flow in the study area, indicating that the habitat selection and gene flow of pine martens may be driven by different factors [17], [25], [31].This may be because gene flow is driven by mating and dispersal events and habitat selection reflects the behaviour of individual organisms to maximize fitness within home ranges (e.g. [102]).

Our results suggest that it is not only forest masses which serve as favourable environments for dispersal. Scrubland, agroforestry mosaics and grassland habitats also potentially favour dispersal, since the correlation increases as, step by step, these environments are included as predictor variables of pine marten gene flow [Land_B(b) <Land_C(b) <Land_D(b) <Land_E(b)<Land_F(b)]. Original causal modeling identified the same pattern and suggested that Land_Eb and Land_Fb for resistance values of 50 and 100 are the most supported models. Likewise, novel reciprocal causal modeling highlighted similar results, identifying Land_Fb100 as the uniquely supported model, due to its greater discriminatory power [84].

These results are in consonance with recent ecological studies of European pine martens, based on radio tracking, which provide new data substantially differing from traditional descriptions in the scientific literature as strictly forest dependant species [48], [51], [55]. These studies show that martens are not exclusively confined to extensive forest patches but that they also use other patches including scrubland and agroforestry mosaics [48], [51], [54], [55]. Indeed, the inclusion of scrub habitat in marten home ranges is likely to be related to its role in the connectivity of forest habitats [48], [55]. In the same way, the improvement in correlation obtained by including pastures and meadows indicates that the species does not always avoid crossing these open spaces areas when there is forest habitat in the immediate vicinity as has been previously suggested by radiotracking data [48]. This is precisely the case in the area under study, where pastures and meadows are typically found in the immediate vicinity of forest. However, the inclusion of homogeneous croplands reduces the correlation between genetic distance and effective distance, suggesting that zones with intensive agriculture potentially impede species dispersal. This could be due to the scarcity of natural vegetation in these zones and the distance separating them from forest in the study area [41].

Additionally, models that increase the barrier effect of major roads and urban areas leads to a substantial improvement in the correlation between genetic distances and cost distances. The correlation with models Land_Ab-Land_Fb was greater than that obtained with models Land_A-Land_F, and the uniquely supported model in reciprocal causal modeling included the barrier effect (Land_Fb100). This suggests that the potential barrier effect of these land uses could have a synergic effect within a fragmented landscape, decreasing the gene flow due to road avoidance behaviour and/or road mortality [103]–[106].

Similar landscape genetics studies have also been conducted on other forest dependant *Martes* sp., providing contrasting results regarding landscape effects on gene flow [25], [32], [107]–[110]. Similar to the results found in this study, Broquet et al. [32], found that American marten (*Martes americana*) dispersal in Ontario is impeded by the loss and fragmentation of suitable habitat. Wasserman et al. [25] showed that gene flow in the Northern Idaho American marten population is driven by a gradient function of elevation, which was a proxy for snowpack, with marten avoiding lower elevations and dispersing in mid to high elevation montane forests. In contrast, Koen et al. [110] found that marten dispersal across Ontario can best be described as neighbour-mating with no directional bias caused by forest-management induced landscape structure, resulting in a pattern of isolation by distance, suggesting that Ontario landscape is well connected with respect to suitable marten habitat. These contrasting landscape hypotheses governing gene flow could be explained by the different limiting factors that could be acting in each of landscape under study [22].

Even though no previous individual-based landscape genetics data was available for the pine marten, Mergey et al. [58] found that genetic diversity is not associated with habitat fragmentation metrics in France, in spite of the existence of a high degree of forest fragmentation in the studied marten populations. However, this result does not demonstrate that the pine marten gene flow is not affected by forest fragmentation processes. Thus, a more detailed individual-based landscape genetics analysis (Larroque et al. Unpublished data), could provide better insights into the landscape processes governing gene flow and an interesting comparative framework with Spanish pine marten populations.

### Empirical evaluation of ecological network resistance maps through landscape genetics

Maps of ecological corridors are commonly used in land use planning, but unfortunately are more often the product of expert opinion rather than empirical data [9], [10]. Thus, using landscape genetic analysis, we could partially solve this limitation by studying the gene flow of a target species with regards to the resistance maps used to design the ecological networks [11], [12]. Here, the parameterization found in the resistance map which was used to design the regional corridors linking forest protected areas of the Basque Country (north Spain) [41] was adequate to explain pine marten gene flow, with one of the highest partial Mantel r value (r =  0.145) of all the evaluated models. Based on reciprocal causal modeling only Land_Fb100, Land_Eb100, Land_Fb50 and Land_Eb50 were supported independently of EN. Even though Land_Fb100 better explains pine marten gene flow, the high mantel correlation value between the cost distances for Land_Fb100 and EN (Mantel r = 0.9362 p<0.001) suggests EN is a good proxy. This indicates that the EN model used to develop regional connectivity networks among protected areas [41] likely performs very well as a surrogate for landscape resistance for pine marten.

Thus, the resistance map with which the regional ecological network was originally designed in the Basque Country (EN), appears to have high congruence with one of its official target species at regional scale [41]. This is a welcome finding, given that most past evaluations of expert-derived resistance values found that they performed poorly in comparison to empirically optimized models [18], [24]. Given the importance of pine marten as a bio-indicator of species associated with natural vegetation [41], our results emphasize the importance of incorporating regional corridors into land use planning and management to preserve landscape connectivity for forest dwelling species.

### The influence of resistance values and logarithmic transformations to detect landscape genetic relationships

Since our ability to detect the effects of landscape structure on genetic differentiation depends on both the landscape features used and the relative costs of each feature, different resistance values could provide different results [17], [19], [83]. Previous studies have found that the degree of contrast in resistance to gene flow in habitat as compared to non-habitat could affect whether or not a given landscape configuration will significantly affect genetic differentiation [22]. We found an increasing reliability of predictions as resistance contrast increased, with several models only supported by 25, 50 and 100 resistance values and unimodal peak of support for 50 and 100 resistance values. The hypothesis that was uniquely supported by reciprocal causal modeling indicated that non-habitat was 100 times more resistant than habitat, and anthropogenic barriers may impart additional resistance as high as 1000 times that of optimal habitat.

Some landscape genetic studies have found that the untransformed geographic distances perform better than logarithmic transformation (e.g. [24], [30]), while most previously studies used transformed distances without any evaluation. However, the relationship between cost distances and genetic distances is highly dependant on the study area and the focal species. For example, at small extents the relationship between cost and genetic distances is nearly linear and the untrasformed correlations may fit the data better [24], [30]. However, when the study area is large in extent relative to the dispersal ability of the species, as in the present study, the relationship between cost distance and genetic distance will be nonlinear and the logarithm transform will improve fit. Thus, taking into account the potential bias due to an incorrect use of transformations, we propose that future landscape genetics should evaluate the unimodality of support among the hypotheses as a means to determine the degree to which the transformation improves the analysis.

## Conclusions

This paper presents a comprehensive individual-based landscape genetic analysis of the European pine marten, and the first formal use of landscape genetics to evaluate the effectiveness of regional ecological networks. We compared results from several methods of model selection and found that ranking based on Mantel r or partial Mantel r, the unimodality of support in the hypothesis cube, causal modeling and reciprocal causal modeling all identified the same best models of landscape resistance for European pine marten in northern Spain. Reciprocal causal modeling appeared to provide the strongest differentiation among hypotheses and enabled the identification of a single, independently supported model. Gene flow of European pine marten is facilitated by natural land cover, such as forest, scrublands and pastures and meadows, and is resisted by anthropogenic land uses and linear barriers such as major roads. We confirm that the resistance map used to develop the regional ecological network in the Basque Country is a close surrogate to the empirically optimized resistance model for marten.

## Supporting Information

Figure S1 **Mantel Correlation values for a) Land_A-Land_G and b) Land_Ab-Land_Gb models for the 4 different cost values evaluated on the log transformed cost distances.**
(TIF)

Figure S2 **Partial Mantel Correlation values for a) Land_A-Land_G and b) Land_Ab-Land_G models for the 4 different cost values evaluated on the log transformed cost distances.**
(TIF)

Figure S3 **Mantel Correlation values for a) Land_A-Land_G and b) Land_Ab-Land_G models for the 4 different cost values evaluated on the untransformed cost distances.**
(TIF)

Figure S4 **Partial Mantel Correlation values for a) Land_A-Land_G and b) Land_Ab-Land_G models for the 4 different cost values evaluated on the untransformed cost distances.**
(TIF)

Table S1 **Summary of the genetic variability.** Genetic variability of the 15 microsatellite loci multiplexed used in this study. Number of alleles (N_A_), Observed (H_O_) and expected (H_E_) heterozygosities for each locus and for whole data set.. Loci marked with an asterisk deviated from Hardy-Weinberg proportions.
(DOC)

Table S2 **Sample locations and microsatellite data.** Complete genetic profiles (15 microsatellite loci) and the geographic coordinates for the 101 pine marten individuals
(XLS)

Table S3 **Results** **of causal modeling of landscape resistance on genetic distance in European pine marten according to Mantel and partial mantel tests for the untransformed distances.**
(DOC)

File S1 **Ecological network resistance map.** Raw '.asc' file of the EN resistance map from which all the resistance maps evaluated can be produced following the resistance values outlined in Table 1.
(ZIP)
