# Supplementary figures and images for: Landscape Genetics for the Empirical Assessment of Resistance Surfaces: The European Pine Marten (Martes martes) as a Target-Species of a Regional Ecological Network
Source: PLoS One. 2014 Oct 16;9(10):e110552. doi: 10.1371/journal.pone.0110552 (PMC4199733; doi:10.1371/journal.pone.0110552)

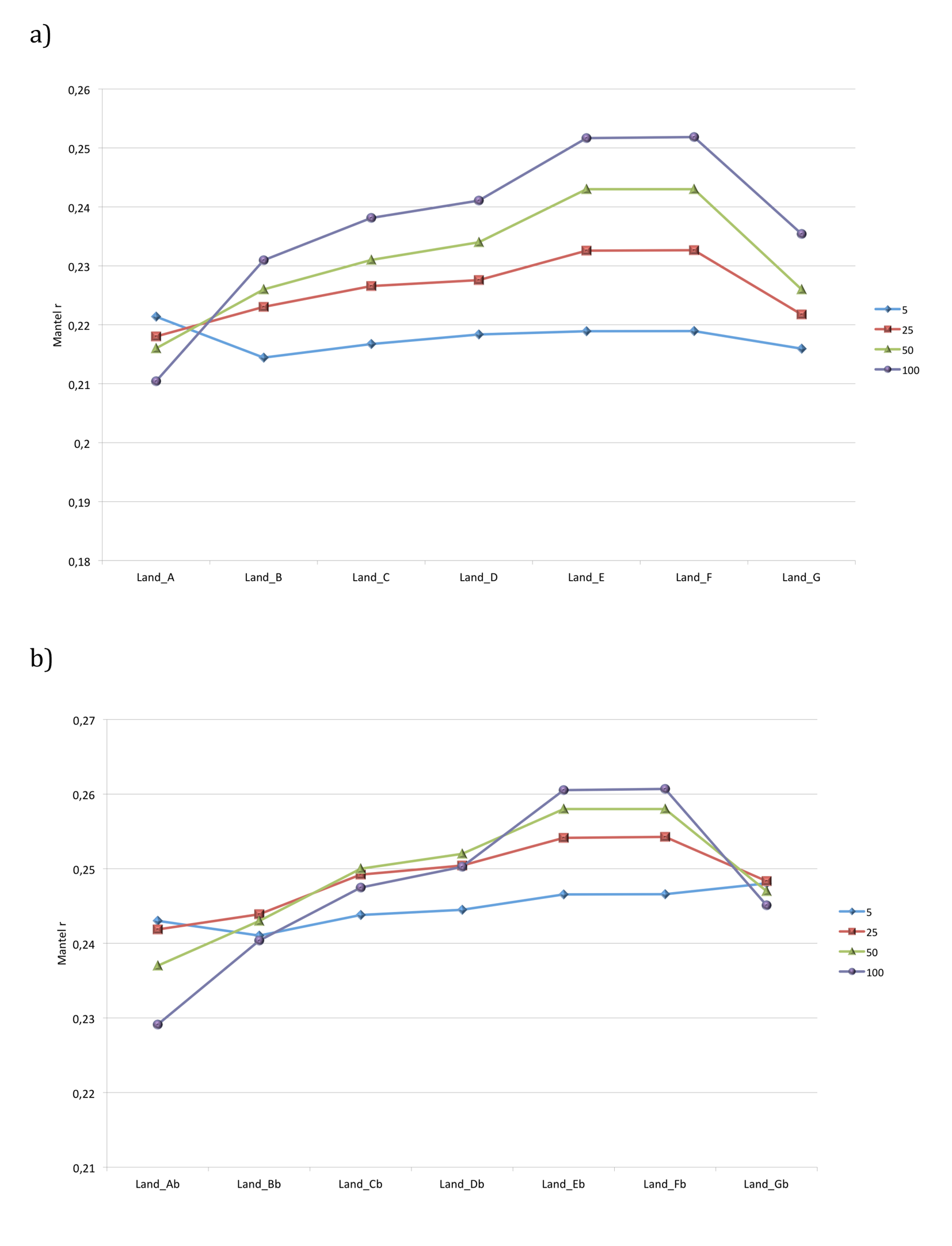

Supplement: Figure S1 — Mantel Correlation values for a) Land_A-Land_G and b) Land_Ab-Land_Gb models for the 4 different cost values evaluated on the log transformed cost distances. (TIF) [file pone.0110552.s001.tif]

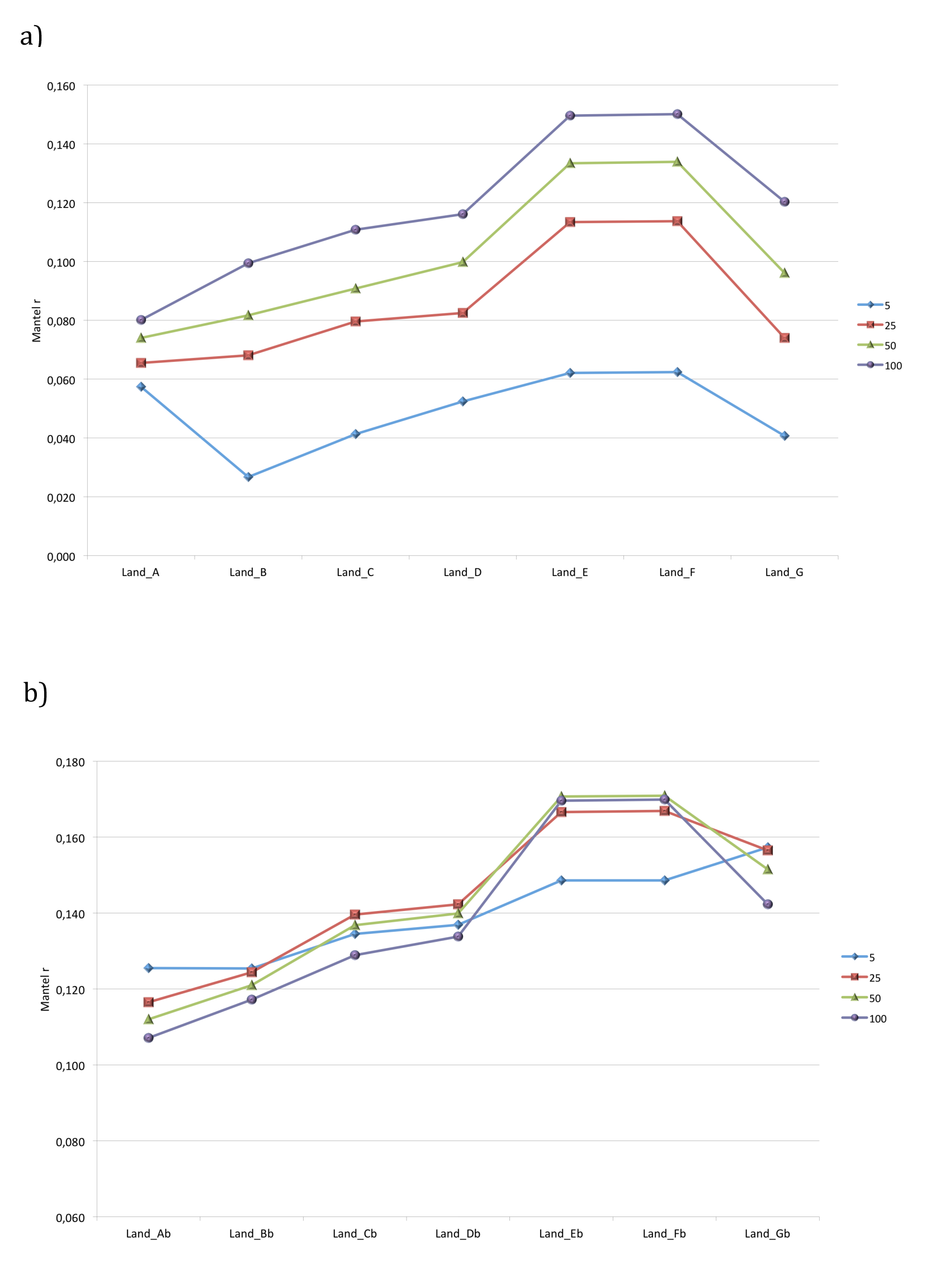

Supplement: Figure S2 — Partial Mantel Correlation values for a) Land_A-Land_G and b) Land_Ab-Land_G models for the 4 different cost values evaluated on the log transformed cost distances. (TIF) [file pone.0110552.s002.tif]

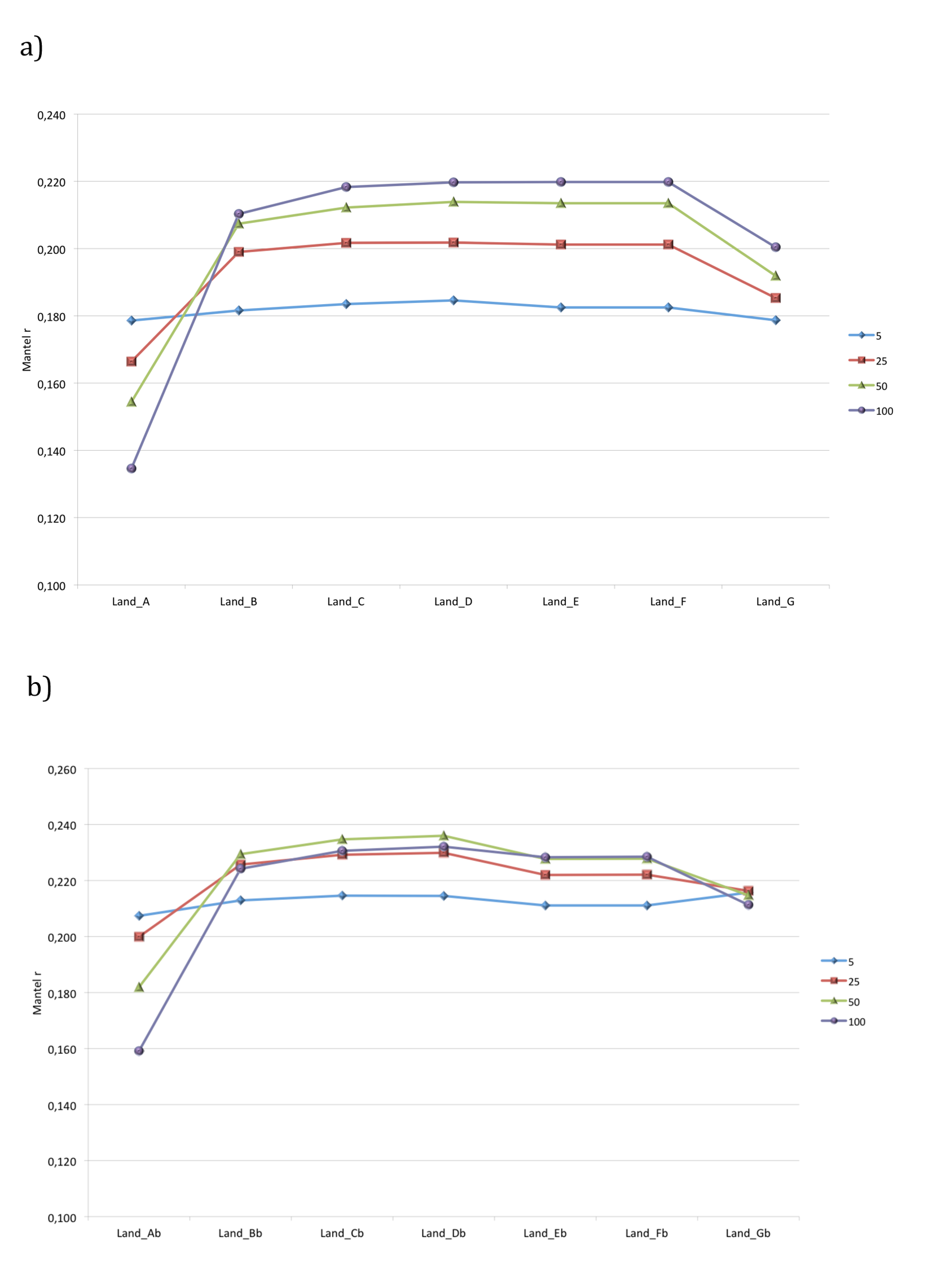

Supplement: Figure S3 — Mantel Correlation values for a) Land_A-Land_G and b) Land_Ab-Land_G models for the 4 different cost values evaluated on the untransformed cost distances. (TIF) [file pone.0110552.s003.tif]

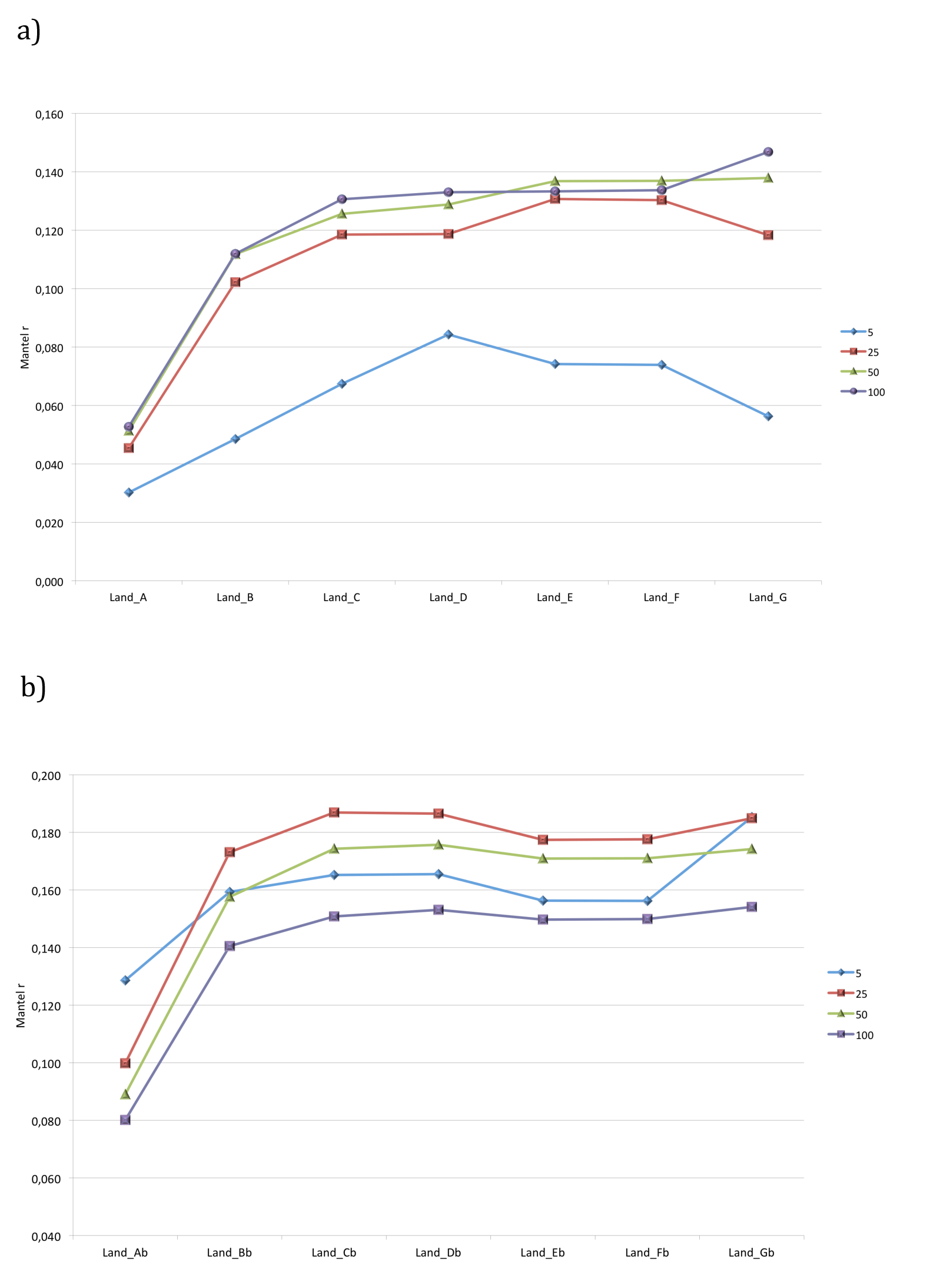

Supplement: Figure S4 — Partial Mantel Correlation values for a) Land_A-Land_G and b) Land_Ab-Land_G models for the 4 different cost values evaluated on the untransformed cost distances. (TIF) [file pone.0110552.s004.tif]
