# Supplementary material for: Landscape Genetics for the Empirical Assessment of Resistance Surfaces: The European Pine Marten (Martes martes) as a Target-Species of a Regional Ecological Network
Source: PLoS One. 2014 Oct 16;9(10):e110552. doi: 10.1371/journal.pone.0110552 (PMC4199733; doi:10.1371/journal.pone.0110552)
Supplement: Table S1 — Summary of the genetic variability. Genetic variability of the 15 microsatellite loci multiplexed used in this study. Number of alleles (NA), Observed (HO) and expected (HE) heterozygosities for each locus and for whole data set.. Loci marked with an asterisk deviated from Hardy-Weinberg proportions. (DOC) [file pone.0110552.s005.doc]

**Table S1. Summary of the genetic variability.** Genetic variability of the 15 microsatellite *loci* multiplexed used in this study. The table includes: number of alleles (N_A_) and observed (H_O_) and expected (H_E_) heterozygosities for each locus and for whole data set. Loci marked with an asterisk deviated from Hardy-Weinberg proportions.

| **MULTIPLEX** | **Locus** | **N_A_** | **H_E_** | **H_O_** |
| --- | --- | --- | --- | --- |
| **MULT_1** | Gg-7 | 5 | 0.66 | 0.59 |
|  | Ma-1 | 4 | 0.56 | 0.45 |
|  | MP0059 | 5 | 0.65 | 0.49 |
|  | MP0188* | 3 | 0.46 | 0.35 |
| **MULT_2** | Lut-453 | 3 | 0.50 | 0.57 |
|  | Mel-1 | 5 | 0.65 | 0.69 |
|  | Mel-10 | 5 | 0.66 | 0.57 |
| **MULT_3** | Lut-435* | 7 | 0.42 | 0.28 |
|  | Ma-19 | 4 | 0.54 | 0.55 |
|  | Mvi-57 | 6 | 0.37 | 0.36 |
|  | Mvi072 | 8 | 0.71 | 0.62 |
| **MULT_4** | Lut-615 | 2 | 0.47 | 0.53 |
|  | Ma-2 | 5 | 0.68 | 0.62 |
|  | Mer41 | 6 | 0.77 | 0.68 |
|  | Mlut-27 | 4 | 0.66 | 0.62 |
| **Mean** |  | **4.13** | **0.58** | **0.53** |
