# Supplementary material for: Landscape Genetics for the Empirical Assessment of Resistance Surfaces: The European Pine Marten (Martes martes) as a Target-Species of a Regional Ecological Network
Source: PLoS One. 2014 Oct 16;9(10):e110552. doi: 10.1371/journal.pone.0110552 (PMC4199733; doi:10.1371/journal.pone.0110552)
Supplement: Table S3 — Results of causal modeling of landscape resistance on genetic distance in European pine marten according to Mantel and partial mantel tests for the untransformed distances. (DOC) [file pone.0110552.s007.doc]

**Table S3.** Results of causal modeling of landscape resistance on genetic distance in European pine marten according to Mantel and partial mantel tests for the untransformed distances.

|  | **Model / Resistance Values** | **1) Simple mantel** | | **Rank** | **2) Partial mantel** | | | **Rank** | **3) Partial Mantel** | | **CMS?** |  | **1) Simple mantel** | | **Rank** | **2) Partial mantel** | | **Rank** | **3) Partial mantel** | | **CMS?** |
| --- | --- | --- | --- | --- | --- | --- | --- | --- | --- | --- | --- | --- | --- | --- | --- | --- | --- | --- | --- | --- | --- |
|  |  | *G*L* | *G*L* |  | *G*L\|Dis* | *G*L\|Dis* | |  | *G*Dis\|L* | G*Dis\|L |  |  | *G*L* | *G*L* |  | *G*L\|Dis* | *G*L\|Dis* |  | *G*Dis\|L* | *G*Dis\|L* |  |
|  |  | *R* | *p* |  | R | p | |  | R | p |  |  | *R* | p |  | R | *p* |  | R | p |  |
| **Binary Landscape Resistance Models (Land_A to Land G)** | **Land_A** *Forest* | | |  |  |  | |  |  |  |  | **Binary Landscape Resistance Models (Land_Ab to Land_Gb)** | **Land_Ab** |  |  |  |  |  |  |  |  |
|  | 5 | *0.179* | *0.0001* | 54 | 0.030 | 0.586 | | 58 | *-0.007* | *0.877* | **N** |  | *0.207* | *0.0001* | 35 | *0.129* | *0.0030* | 37 | *-0.067* | *0.168* | **Y** |
|  | 25 | *0.166* | *0.0019* | 55 | 0.045 | 0.451 | | 57 | *0.047* | *0.317* | **N** |  | *0.200* | *0.0001* | 43 | 0.100 | 0.0854 | 46 | *0.031* | *0.577* | **N** |
|  | 50 | *0.154* | *0.0053* | 57 | 0.051 | 0.404 | | 55 | *0.066* | *0.156* | **N** |  | *0.182* | *0.0008* | 51 | 0.089 | 0.1365 | 47 | *0.077* | *0.134* | **N** |
|  | 100 | *0.135* | *0.0192* | 58 | 0.053 | 0.394 | | 54 | *0.089* | *0.053* | **N** |  | *0.159* | *0.0037* | 56 | 0.080 | 0.1839 | 49 | *0.077* | *0.134* | **N** |
|  | *Mean (*±*SE)* | 0.1585 (±0.0187) | |  | 0.0449 (±0.0103) | | |  |  |  |  |  | 0.1871 (±0.0215) | |  | 0.0994 (±0.0211) | |  |  |  |  |
|  | **Land_B** *Forest + Forestry plantations* | | | | | |  | | | | |  | **Land_Bb** |  |  |  |  |  |  |  |  |
|  | 5 | *0.182* | *0.0001* | 52 | 0.049 | 0.323 | | 56 | *0.011* | *0.764* | **N** |  | *0.213* | *0.0001* | 29 | *0.159* | *0.0001* | 16 | *-0.105* | *0.011* | **Y** |
|  | 25 | *0.199* | *0.0003* | 44 | *0.102* | *0.080* | | 45 | *0.009* | *0.838* | **N** |  | *0.226* | *0.0001* | 13 | *0.173* | *0.0010* | 10 | *-0.099* | *0.063* | **Y** |
|  | 50 | *0.207* | *0.0001* | 36 | *0.112* | *0.058* | | 43 | *0.024* | *0.612* | **N** |  | *0.229* | *0.0001* | 7 | *0.158* | *0.0075* | *17* | *-0.053* | *0.338* | **Y** |
|  | 100 | *0.210* | *0.0001* | 34 | *0.112* | *0.050* | | 39 | *0.040* | *0.380* | **N** |  | *0.224* | *0.0001* | 14 | *0.141* | *0.0209* | *26* | *-0.053* | *0.348* | **Y** |
|  | *Mean (*±*SE)* | 0.1996 (±0.0129) | |  | 0.0936 (±0.0304) | | |  |  |  |  |  | 0.22231 (±0.0071) | |  | 0.1576 (±0.0134) | |  |  |  |  |
|  | **Land_C** *Forest + forestry plantations + Scrublands* | | | | | | | | | | |  | **Land_Cb** |  |  |  |  |  |  |  |  |
|  | 5 | *0.184* | *0.0001* | 48 | 0.067 | 0.161 | | 52 | *-0.006* | *0.863* | **N** |  | *0.215* | *0.0001* | 24 | 0.165 | *0.0002* | *14* | *-0.110* | *0.010* | **Y** |
|  | 25 | *0.202* | *0.0001* | 39 | *0.119* | *0.039* | | 41 | *-0.009* | *0.840* | **Y** |  | *0.229* | *0.0001* | 8 | 0.187 | *0.0001* | *1* | *-0.115* | *0.025* | **Y** |
|  | 50 | *0.212* | *0.0001* | 30 | *0.126* | *0.035* | | 38 | *0.008* | *0.868* | **Y** |  | *0.235* | *0.0001* | 2 | *0.174* | *0.0019* | *8* | *-0.077* | *0.169* | **Y** |
|  | 100 | *0.218* | *0.0001* | 20 | *0.131* | *0.033* | | 34 | *0.021* | *0.648* | **Y** |  | *0.231* | *0.0001* | 5 | 0.151 | *0.0138* | *22* | *-0.077* | *0.169* | **Y** |
|  | *Mean (*±*SE)* | 0.2039 (±0.0152) | |  | 0.01105 (±0.0292) | | |  |  |  |  |  | 0.2273(±0.0088) | |  | 0.1693 (±0.0152) | |  |  |  |  |
|  | **Land_D** *Forest + forestry plantations + scrublands + agroforestry mosaics* | | | | | | | | | | |  | **Land_Db** |  |  |  |  |  |  |  |  |
|  | 5 | *0.185* | *0.0002* | 47 | 0.084 | 0.084 | | 48 | *-0.019* | *0.608* | **N** |  | *0.215* | *0.0001* | 25 | *0.166* | *0.0001* | *13* | *-0.111* | *0.007* | **Y** |
|  | 25 | *0.202* | *0.0001* | 38 | *0.119* | *0.038* | | 40 | *-0.012* | *0.801* | **Y** |  | *0.230* | *0.0001* | 6 | *0.187* | *0.0004* | *2* | *-0.113* | *0.031* | **Y** |
|  | 50 | *0.214* | *0.0001* | 26 | *0.129* | *0.033* | | 36 | *-0.001* | *0.981* | **Y** |  | *0.236* | *0.0001* | 1 | *0.176* | *0.0022* | *7* | *-0.076* | *0.175* | **Y** |
|  | 100 | *0.220* | *0.0001* | 19 | *0.133* | *0.030* | | 32 | *0.017* | *0.721* | **Y** |  | *0.232* | *0.0001* | 3 | *0.153* | *0.0124* | *21* | *-0.076* | *0.177* | **Y** |
|  | *Mean (*±*SE)* | 0.2050 (±0.0155) | |  | 0.01162 (±0.0221) | | |  |  |  |  |  | 0.2281 (±0.0094) | |  | 0.1702 (±0.0143) | |  |  |  |  |
|  | **Land_E** *Forest + forestry plantations + scrublands + agroforestry mosaics + pastures* | | | | | | | | | | |  | **Land_Eb** |  |  |  |  |  |  |  |  |
|  | 5 | *0.183* | *0.0001* | 49 | 0.074 | 0.117 | | 50 | *-0.035* | *0.265* | **N** |  | *0.211* | *0.0001* | 32 | *0.156* | *0.0001* | *18* | *-0.1039* | *0.0088* | **Y** |
|  | 25 | *0.201* | *0.0001* | 40 | *0.131* | *0.024* | | 33 | *-0.061* | *0.132* | **Y** |  | *0.222* | *0.0001* | 16 | *0.177* | *0.0001* | *6* | *-0.1144* | *0.0159* | **Y** |
|  | 50 | *0.214* | *0.0001* | 27 | *0.137* | *0.024* | | 29 | *-0.064* | *0.142* | **Y** |  | *0.228* | *0.0001* | 12 | *0.171* | *0.0011* | *12* | *-0.0899* | *0.0948* | **Y** |
|  | 100 | *0.220* | *0.0001* | 17 | *0.133* | *0.031* | | 31 | *-0.061* | *0.176* | **Y** |  | *0.228* | *0.0001* | 10 | *0.150* | *0.0119* | *24* | *-0.0898* | *0.0909* | **Y** |
|  | *Mean (*±*SE)* | 0.2403 (±0.0164) | |  | 0.1188 (±0.0298) | | |  |  |  |  |  | 0.2223(±0.0080) | |  | 0.1636 (±0.0128) | |  |  |  |  |
|  | **Land_F** *Forest + forestry plantations + scrublands + agroforestry mosaics + pastures + rocky areas* | | | | | | | | | | |  | **Land_Fb** |  |  |  |  |  |  |  |  |
|  | 5 | *0.183* | *0.0001* | 50 | 0.074 | 0.123 | | 51 | *-0.035* | *0.266* | **N** |  | *0.211* | *0.0001* | 33 | *0.156* | *0.0001* | *19* | *-0.104* | *0.010* | **Y** |
|  | 25 | *0.201* | *0.0001* | 41 | *0.130* | *0.021* | | 35 | *-0.062* | *0.134* | **Y** |  | *0.222* | *0.0001* | 15 | *0.178* | *0.0003* | *5* | *-0.115* | *0.017* | **Y** |
|  | 50 | *0.214* | *0.0001* | 28 | *0.137* | *0.021* | | 28 | *-0.064* | *0.140* | **Y** |  | *0.228* | *0.0001* | 11 | *0.171* | *0.0019* | *11* | *-0.090* | *0.095* | **Y** |
|  | 100 | *0.220* | *0.0001* | 18 | *0.134* | *0.030* | | 30 | *-0.061* | *0.184* | **Y** |  | *0.229* | *0.0001* | 9 | *0.150* | *0.0120* | *23* | *-0.090* | *0.094* | **Y** |
|  | *Mean (*±*SE)* | 0.2403 (±0.0164) | |  | 0.1187 (±0.0300) | | |  |  |  |  |  | 0.2224(±0.0080) | |  | 0.1637 (±0.0128) | |  |  |  |  |
|  | **Land__G** *Forest + forestry plantations + scrublands + agroforestry mosaics + pastures + rocky areas + croplands* | | | | | | | | | | |  | **Land_Gb** |  |  |  |  |  |  |  |  |
|  | 5 | *0.179* | *0.0001* | 53 | 0.056 | 0.065 | | 53 | *-0.018* | *0.351* | **N** |  | *0.2157* | *0.0001* | 22 | *0.185* | *0.0001* | *3* | *-0.137* | *0.001* | **Y** |
|  | 25 | *0.185* | *0.0001* | 46 | *0.118* | *0.0003* | | 42 | *-0.039* | *0.125* | **Y** |  | *0.2163* | *0.0001* | 21 | *0.185* | *0.0001* | *4* | *-0.136* | *0.001* | **Y** |
|  | 50 | *0.192* | *0.0001* | 45 | *0.138* | *0.0001* | | 27 | *-0.051* | *0.077* | **Y** |  | *0.2147* | *0.0001* | 23 | *0.174* | *0.0001* | *9* | *-0.124* | *0.001* | **Y** |
|  | 100 | *0.200* | *0.0001* | 42 | *0.147* | *0.0001* | | 25 | *-0.063* | *0.043* | **Y** |  | *0.2113* | *0.0001* | 31 | *0.154* | *0.0002* | *20* | *-0.124* | *0.001* | **Y** |
|  | *Mean (*±*SE)* | 0.1891 (±0.0093) | |  | 0.1148 (±0.0408) | | |  |  |  |  |  | 0.2145 (±0.0022) | |  | 0.1746 (±0.0146) | |  |  |  |  |
|  | **ENnb** |  |  |  |  |  | |  |  |  |  |  | ***EN*** |  |  |  |  |  |  |  |  |
|  |  | *0.206* | *0.0001* | 37 | *0.107* | *0.007* | | 44 | *-0.003* | 0.947 | **Y** |  | *0.2316* | *0.0001* | *4* | *0.145* | *0.0042* | *15* | *-0.0563* | *0.3173* | **Y** |

Model definitions according to Table 1. There are 3 Mantel tests comprising causal modeling: (1) G*L—simple Mantel test between the candidate model and genetic distance; (2) G*L|Dis—partial Mantel test between the candidate model and genetic distance, partialling out Euclidean distance;(3) G*D|L—partial Mantel test between the Euclidean and genetic distance, partialling out the candidate model. For a candidate model to be supported tests (1) and (2) must be significant, while test (3) must be negative or non-significant. Mantel tests meeting each criterion are italicized. Ranking of each model according to Mantel and partial Mantel r values is included. CMS? indicates if the model is supported within the causal modeling framework (Y) or not (N).
